# Analysis of DNA methylation landscape reveals the roles of DNA methylation in the regulation of drug metabolizing enzymes

**DOI:** 10.1186/s13148-015-0136-7

**Published:** 2015-09-28

**Authors:** Wataru Habano, Kohei Kawamura, Natsuki Iizuka, Jun Terashima, Tamotsu Sugai, Shogo Ozawa

**Affiliations:** Department of Pharmacodynamics and Molecular Genetics, School of Pharmacy, Iwate Medical University, 2-1-1 Nishitokuta, Yahaba-Cho, Shiwa-Gun 028-3694 Japan; Department of Diagnostic Pathology, School of Medicine, Iwate Medical University, Iwate, Japan

**Keywords:** DNA methylation mapping, Differences in drug metabolism, Integrative analysis, Alternative splicing

## Abstract

**Background:**

Drug metabolizing enzymes (DMEs) exhibit dramatic inter- and intra-individual variability in expression and activity. However, the mechanisms determining this variability have not been fully elucidated. The aim of this study was to evaluate the biological significance of DNA methylation in the regulation of DME genes by genome-wide integrative analysis.

**Results:**

DNA methylation and mRNA expression profiles of human tissues and hepatoma cells were examined by microarrays. The data were combined with GEO datasets of liver tissues, and integrative analysis was performed on selected DME genes. Detailed DNA methylation statuses at individual CpG sites were evaluated by DNA methylation mapping. From analysis of 20 liver tissues, highly variable DNA methylation was observed in 37 DME genes, 7 of which showed significant inverse correlations between DNA methylation and mRNA expression. In hepatoma cells, treatment with a demethylating agent resulted in upregulation of 5 DME genes, which could be explained by DNA methylation status. Interestingly, some DMEs were suggested to act as tumor-suppressor or housekeeper based on their unique DNA methylation features. Moreover, tissue-specific and age-dependent expression of UDP-glucuronosyltransferase 1A splicing variants was associated with DNA methylation status of individual first exons.

**Conclusions:**

Some DME genes were regulated by DNA methylation, potentially resulting in inter- and intra-individual differences in drug metabolism. Analysis of DNA methylation landscape facilitated elucidation of the role of DNA methylation in the regulation of DME genes, such as mediator of inter-individual variability, guide for correct alternative splicing, and potential tumor-suppressor or housekeeper.

**Electronic supplementary material:**

The online version of this article (doi:10.1186/s13148-015-0136-7) contains supplementary material, which is available to authorized users.

## Background

Inter-individual differences in responses to drug therapy vary widely. Such differences are due in part to variable pharmacokinetics, which can be explained by diversity in genes affecting drug absorption, distribution, metabolism, and excretion (ADME) [1]. For example, genetic polymorphisms in cytochrome P450 (CYP) genes, such as *CYP2C9*, *CYP2C19*, and *CYP2D6*, affect drug metabolizing activity and lead to different drug responses [2–4]. Inter-individual differences are also observed in *CYP3A4* expression and activity [5, 6] but are rarely associated with any detectable polymorphisms in the *CYP3A4*. Additionally, UDP-glucuronosyltransferase 1A1 (*UGT1A1*) mutant genotypes *28 and *6 are related to poor glucuronidation activity and have been shown to act as useful indictors of adverse reactions to cancer chemotherapy with irinotecan (CPT-11) [7, 8]. However, some individuals who do not have these mutant genotypes also suffer from adverse reactions to irinotecan [9].

Intra-individual differences are also involved in the regulation of ADME-related genes. For example, the expression and metabolizing activity of UGT1A isoforms vary among tissues and during normal development [10, 11]. The *UGT1A* locus encodes nine functional isoforms through an exon sharing mechanism in which the transcripts of individual first exon cassettes are spliced to exons 2–5, leading to the expression of individual UGT1A isoforms [12]. Although the tissue-specific and age-dependent expressions of the UGT1A isoforms are important determinants of drug efficacy and adverse reactions, the regulatory mechanisms involved in UGT1A expression cannot be explained by genetic polymorphisms. Thus, the mechanisms underlying inter- and intra-individual differences in responses to drug therapy have not been fully elucidated.

In order to examine these mechanisms, we investigated the involvement of epigenetics, the mechanism of heritable changes in gene regulation without DNA sequence alteration. We previously found that epigenetic mechanisms, such as DNA methylation, are involved in the regulation of drug metabolizing enzymes (DMEs) in colon cancer cells [13, 14]. The *CYP1B1* and *CYP3A4* genes were shown to be transcriptionally upregulated by treatment with a demethylating agent, and such upregulation could be explained by hypermethylation at CpG sites located in the 5′-promoters of the *CYP1B1* and pregnane X receptor (*PXR*) genes. To date, more than 50 ADME-related genes have been reported as targets for epigenetic regulation [15, 16]. Although these ADME-related genes were found to be aberrantly regulated by epigenetic mechanisms in tumor cells, most ADME-related genes have been discovered unexpectedly by multiple independent studies during the search for tumor suppressor genes. Therefore, we still have a limited understanding of the biological significance of epigenetic mechanisms in the regulation of ADME-related genes.

In the present study, we examined global DNA methylation and mRNA expression profiles of human tissues and hepatoma cell lines using microarray platforms. These two omic datasets were combined with similar corresponding datasets derived from the Gene Expression Omnibus (GEO) database at the National Center for Biotechnology Information (NCBI). In order to evaluate the significance of DNA methylation in the regulation of DME genes, we examined which DME genes were regulated by DNA methylation in normal liver tissues and hepatoma cells, whether the tissue-specific and age-dependent expression of UGT1A isoforms could be regulated by DNA methylation, and whether DNA methylation profiles could be used to elucidate the specific roles of DME genes. To this end, we performed DNA methylation mapping to determine the methylation levels and variations at individual CpG sites in relation to the structure of individual DME genes. Recent studies demonstrated that global and detailed analysis of dynamic DNA methylation profiles facilitated the finding of the informative fraction of CpG sites [17, 18]. Therefore, we would be able to identify the role of DNA methylation of DME genes by analysis of DNA methylation landscapes.

## Results

### DNA methylation profiles of DME genes in human tissues

**Fig. 1:**
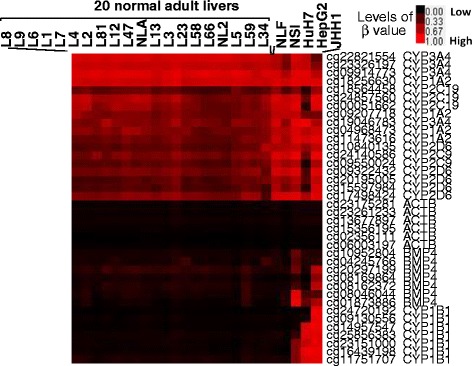
Heat maps of the DNA methylation profiles of the representative 6 *CYP* and 2 control genes by cluster analysis. The top of the map indicates samples from 20 normal adult livers (L1 to L81, NLA and NL2), 1 fetal liver (NLF), 1 adult small intestine (NSI), and 3 hepatoma cell lines. The right of the map indicates the target ID and gene name (*CYP1A2*, *CYP1B1*, *CYP2C9*, *CYP2C19*, *CYP2D6*, *CYP3A4*, *ACTB*, and *BMP4*) examined for individual CpG sites. The representative methylation profiles of individual genes were selected by excluding profiles showing the similar patterns each other. Therefore, the hierarchical similarity tree was not shown in the map. Higher DNA methylation levels are shown in *red*, while lower DNA methylation levels are shown in *black*

**Fig. 2:**
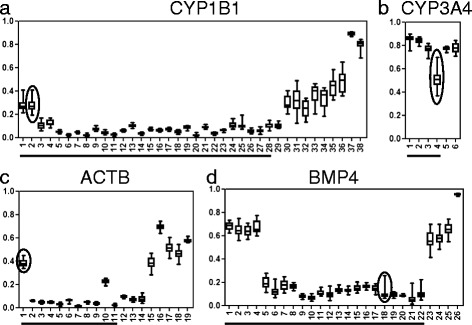
Representative results of DNA methylation mapping for 20 liver tissues. In each panel, the horizontal axis indicates the positions of CpG sites arranged in the 5′ to 3′ direction. The 5′ regulatory region (TSS1500, TSS200, or 5′UTR) is underlined. The vertical axis indicates the *β* value, with the variations expressed as box-and-whisker plots. The maximum *β*_R_ value within the 5′ regulatory region is indicated by a circle for each gene. **a** *CYP1B1*, **b** *CYP3A4*, **c** β-actin (*ACTB*), and **d** bone morphogenetic protein 4 (*BMP4*)

**Fig. 3:**
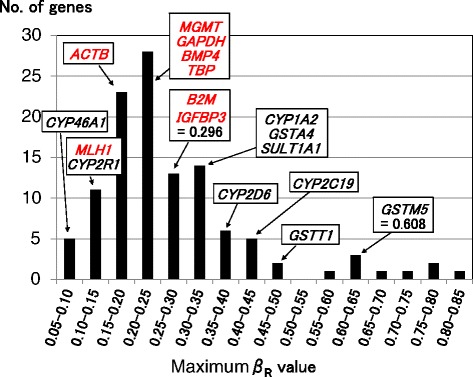
Distribution of maximum *β*_R_ values of the DME and control genes. The maximum *β*_R_ values of the eight control genes colored in *red* were relatively low (≤0.296). We defined highly variable DNA methylation status as a *β*_R_ value of more than 0.296. The *β*_R_ values of representative DME genes are also shown in the histogram. *ACTB* actin, beta, *B2M* β2 microglobulin, *BMP4* bone morphogenetic protein 4, *GAPDH* glyceraldehyde-3-phosphate dehydrogenase, *IGFBP3* insulin-like growth factor binding protein 3, *MGMT O*-6-methylguanine-DNA methyltransferase, *MLH1* mutL homologue 1, *TBP* TATA box-binding protein

We examined DNA methylation profiles of two adult liver tissues (NLA and NL2), fetal liver tissue (NLF), adult small intestinal tissue (NSI), and three hepatoma cell lines (HepG2, HuH7, and JHH1) by HumanMethylation450 Bead Chip. These data were combined with GEO-registered datasets of 18 healthy adult liver tissues. The levels of DNA methylation for more than 480,000 CpG sites were determined as the *β* values (0 < β < 1). Cluster analysis demonstrated that methylation levels of DME genes, including 55 *CYP* genes and 62 phase II DME genes, differed markedly among distinct tissue groups. The representative profiles of six CYPs (*CYP1A2*, *CYP1B1*, *CYP2C9*, *CYP2C19*, *CYP2D6*, and *CYP3A4*), and two controls (*ACTB* and *BMP4*) were shown in Fig. 1. Although the 20 adult liver tissues were derived from two different race populations (2 Chinese and 18 German), they exhibited similar DNA methylation profiles. However, some DME genes such as *CYP1A2*, *CYP2C9*, *CYP2C19*, *CYP2D6*, and *CYP3A4* showed relatively variable methylation statuses, compared to *CYP1B1* and two control genes. To examine the landscape of DNA methylation in more detail, we performed the DNA methylation mapping of individual DME genes (Fig. 2 and Additional file 1: Figure S1). For each CpG site, the range of variation was defined as a *β*_R_ value, which was calculated from the difference between the highest and lowest *β* values among the 20 livers. Next, for all CpG sites located in the 5′ regulatory region (defined as TSS1500, TSS200, or 5′UTR in the array platform), the maximum *β*_R_ value was used for estimation of the degree of inter-individual differences in DNA methylation. The distribution of maximum *β*_R_ values (degrees of variation) of the genes is shown as a histogram in Fig. 3. We expected that the control genes would show the least variation because the expression of these genes should be tightly regulated by DNA methylation on their promoter CpG islands, as reported by Edgar et al. [19]. Therefore, these genes were used as negative controls of inter-individual variation. We finally identified 37 (32 %) DME genes with significantly variable DNA methylation statuses, for which the maximum *β*_R_ values were more than those of all eight control genes (*ACTB*, *B2M*, *GAPDH*, *TBP*, *BMP4*, *IGFBP3*, *MLH1*, and *MGMT*) (maximum *β*_R_ > 0.296).

### DME genes regulated by DNA methylation in adult livers

**Fig. 4:**
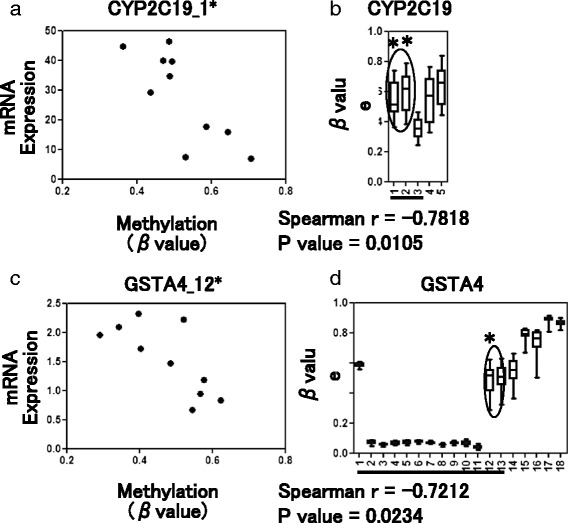
Representative results of correlation analysis. A significant inverse correlation between DNA methylation level (*β* value) and mRNA expression level was detected for the *CYP2C19* (**a**) and *GSTA4* (**c**) genes (*p* < 0.05, Spearman’s rank correlation test). The level of mRNA expression, shown as the vertical axis, was normalized to the expression level of *ACTB* (set as 100 %). CpG sites with significant correlations are indicated by *asterisks* in methylation mapping for the *CYP2C19* (**b**) and *GSTA4* (**d**) genes. Additionally, CpG sites with *β*_R_ values of more than 0.296 are indicated by *circles*

Transcript datasets were also registered for 10 of the 18 liver tissues examined for DNA methylation analysis. We analyzed the mRNA expression profiles of DME genes in these 10 adult livers and detected the highest expression of *CYP2E1* and *CYP3A4* genes, consistent with a previous report [20] (Additional file 2: Figure S2A). In addition, the majority of the DME genes exhibited variable levels of mRNA expression compared to the housekeeping genes (with coefficients of variation (CVs) of more than 17.1 %; Additional file 2: Figure 2B). We found inverse correlations between mRNA expression and DNA methylation levels in seven DME genes (*CYP1A2*, *CYP2C19*, *CYP2D6*, *GSTA4*, *GSTM5*, *GSTT1*, and *SULT1A1*). In particular, the *CYP2C19*, *GSTA4*, and *GSTM5* genes had CpG sites that simultaneously showed inverse correlations and highly variable methylation statuses (*β*_R_ > 0.296; Fig. 4 and Additional file 3: Figure S3).

### DME genes regulated by DNA methylation in three hepatoma cell lines

**Fig. 5:**
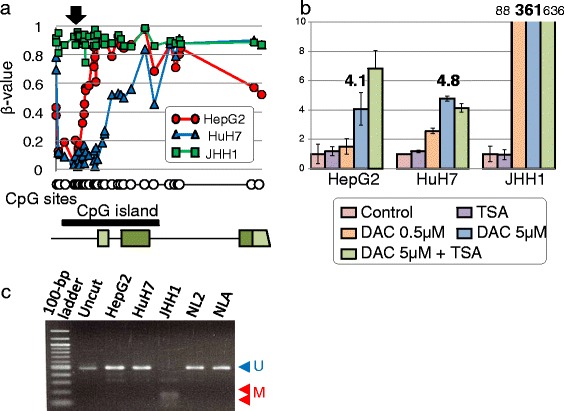
Validation of mRNA expression and DNA methylation of the *CYP1B1* gene in hepatoma cells. **a** The level of DNA methylation was mapped on the *CYP1B1* gene locus in the three hepatoma cell lines. The gene structure and location of the CpG sites are shown in the *lower panel*. **b** The levels of *CYP1B1* mRNA were detected by quantitative real-time PCR in hepatoma cells treated with TSA and/or DAC. The vertical axis indicates the mRNA level of treated cells relative to paired control cells without treatment. Each column represents the mean ± SD (*n* = 3). **c** The *CYP1B1* gene methylation status of the three control cell lines and adult liver tissues was examined by COBRA assay. DNA fragments cleaved by TaqI digestion represent methylated DNA (*M*), while noncleaved fragments represent unmethylated DNA (*U*)

Cluster analysis revealed that the three hepatoma cell lines showed clearly distinct methylation profiles compared to normal liver tissues (Fig. 1). Aberrant DME gene methylation detected in hepatoma cells is summarized in Additional file 4: Figure S4. We defined a hypermethylated or hypomethylated CpG site as having a *β* value of more than 0.5 for comparisons between hepatoma cell lines and normal liver (NL2). This cutoff value was determined by validation analysis, as described below. Using this criterion, 36 DME genes had hypermethylated CpG sites within the 5′ regulatory region in at least one hepatoma cell line. To determine whether the hypermethylation observed in hepatoma cells was associated with downregulation of the DME gene, DNA methylation was reversed by treatment with 5-aza-2′-deoxycytidine (DAC), and mRNA expressions were examined using a SurePrint G3 Human Gene Expression 8 × 60K v2 microarray. As a result, 44 (38 %) DME genes showed upregulation by more than twofold following DAC treatment (Additional file 5: Figure S5). Therefore, these DME genes were downregulated by specific DNA methylation events. Among these DME genes, the downregulation of *CYP1B1*, *CYP8B1*, *GSTM2*, *GSTP1*, *UGT2B15*, and *UGT3A2* in hepatoma cells could be explained by DNA methylation status. The results of array-based DNA methylation and mRNA expression analyses were examined for validity using combined bisulfite restriction analysis (COBRA) and quantitative real-time PCR assays, respectively. The criterion for judging hypermethylation was determined by the results of the tumor suppressor genes used as positive controls (Additional file 6: Figure S6). By using a cutoff value of *β* > 0.5, we identified five DME genes (*CYP1B1*, *CYP8B1*, *GSTM2*, *GSTP1*, and *UGT3A2*) that were regulated by DNA methylation in hepatoma cells. Representative results for the *CYP1B1* gene are shown in Fig. 5.

### DNA methylation and alternative splicing of UGT1A isoforms

**Fig. 6:**
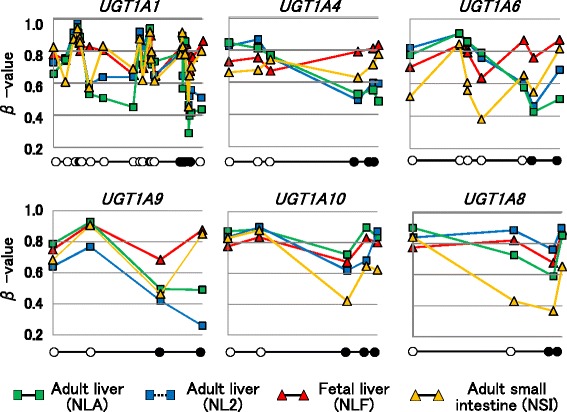
Representative results of DNA methylation mapping of *UGT1A* isoforms. The levels of the DNA methylation of hepatic-type (*UGT1A1*, *UGT1A4*, *UGT1A6*, and *UGT1A9*) and intestinal-type (*UGT1A10* and *UGT1A8*) genes were examined in adult liver tissues (NLA and NL2), fetal liver tissue (NLF), and adult small intestinal tissue (NSI). In each panel, the *open or closed circles located on the horizontal axis* indicate the positions of CpG sites arranged in the 5′ to 3′ direction according to the relative distance of each CpG site. The *closed circles* indicate CpG sites in which tissue-specific expression could be explained by the DNA methylation status

Next, the transcript levels of *UGT1A* isoforms were examined in different tissues using quantitative real-time PCR. The tissue-specific expression profiles shown in Additional file 7: Figure S7 were similar to the results of a previous study [10–12]. The *UGT1A* genes were then classified into two major groups according to the tissues in which they were dominantly expressed: hepatic type (i.e., *UGT1A1*, *UGT1A3*, *UGT1A4*, *UGT1A6*, and *UGT1A9*) and intestinal type (i.e., *UGT1A5*, *UGT1A7*, *UGT1A8*, and *UGT1A10*). Although the level of *UGT1A8* expression in adult livers was somewhat higher than that in small intestines, *UGT1A8*, unlike the other hepatic-type *UGT1A*s, showed relatively high expression in the small intestine as well. Therefore, we classified *UGT1A8* as an intestinal-type gene. DNA methylation mapping on the *UGT1A* locus revealed that DNA methylation status was largely variable among different tissues (Additional file 8: Figure S8). We focused on the first exon of each isoform and found that the hepatic-type genes *UGT1A1*, *UGT1A4*, *UGT1A6*, and *UGT1A9* tended to be methylated at higher levels in the small intestine than in the liver (Fig. 6). In order to perform statistical analysis, moreover, DNA methylation status in individual CpG sites were also compared between 18 adult livers and one normal small intestine (NSI) or one normal fetal liver (NLF). We further focused on the CpG sites most proximal to each transcription start site (excluding more distal CpG sites of TSS1500). In hepatic-type UGT1A genes, as a result, we found that *β* values of the NSI and NLF were statistically higher than medians of 18 individual *β* values of normal adult livers (Additional file 9: Table S1). In contrast, the methylation levels of the intestinal-type genes *UGT1A10* and *UGT1A8* were relatively higher in the liver than in the small intestine. This suggested that DNA methylation status around the first exon determined the splicing isoforms of the *UGT1A* gene and led to tissue-specific expression. In addition, higher levels of DNA methylation were observed in fetal livers than in adult livers. This tendency was found in hepatic-type genes rather than intestinal-type genes, implying that the low levels of expression observed in the fetal liver may result from downregulation by DNA methylation.

### Classification of DME genes based on their DNA methylation landscape

**Fig. 7:**
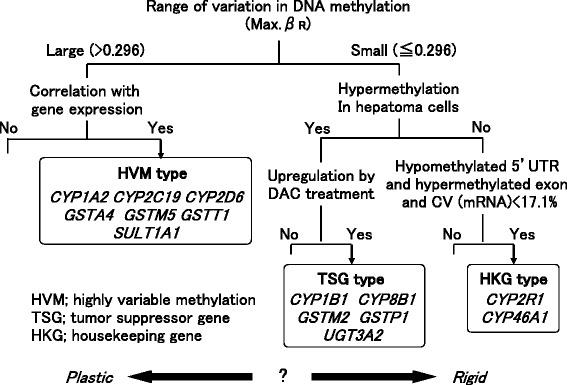
Classification of DME genes based on the corresponding DNA methylation landscape

Interestingly, DME genes had unique features of the DNA methylation landscape (Fig. 1), which could be classified into at least three groups, as summarized in Fig. 7. The first group showed highly variable methylation among normal livers (maximum *β*_R_ > 0.296) and inverse correlations with mRNA expression. DME genes in this first group may be candidates for explaining inter-individual variations in drug metabolizing activity; we classified genes in this group as the highly variable methylation (HVM) type. Genes in the second group, similar to the *CYP1B1* gene, showed stable methylation statuses among normal livers but were hypermethylated in tumor cells. These DNA methylation features were similar to those of tumor suppressor genes (i.e., the TSG type). The last group retained low levels of methylation in both normal and tumor livers, suggesting these genes may act as housekeeping genes. Typical housekeeping genes, such as *ACTB* and *GAPDH*, had similar features of methylation mapping and expression profiles in our study. For example, the level of DNA methylation was highly stable and low in the 5′ regulatory region (hypomethylated 5′UTR) but considerably high within the gene body (hypermethylated exons). In addition, the mRNA expression of the housekeeping genes was stable among normal livers, with CVs of less than 17.1 %. Although it was difficult to fulfill all these criteria, we found two DME genes (*CYP2R1* and *CYP46A1*) that had characteristics similar to those of housekeeping genes (i.e., HKG type).

## Discussion

We used GEO datasets previously registered elsewhere [21] and combine these datasets with our original data. We demonstrated that genome-wide integrative analysis using GEO datasets was a powerful tool for identification of novel roles of DNA methylation. We cannot rule out the possibility of batch effects, which are the technical artifacts such as laboratory conditions, experiment time, reagent lots, and/or laboratory personnel differences [22]. However, the datasets obtained from the same laboratory were used for the study of methylation mapping and correlation analysis. Therefore, we think that such effect was reduced to the lowest level in our study.

The DNA methylation statuses of DME genes varied among normal liver tissues. We found seven DME genes (i.e., *CYP1A2*, *CYP2C19*, *CYP2D6*, *GSTA4*, *GSTM5*, *GSTT1*, and *SULT1A1*) that showed significant inverse correlations between DNA methylation and mRNA expression levels. Some other DME genes also tended to show such correlations, but statistical significance was not reached. These results suggested that a small but significant fraction of DME genes were transcriptionally regulated by DNA methylation, resulting in different mRNA expression levels among individuals. Further investigations with larger sample sizes may allow for identification of additional targets for DNA methylation. Recent studies demonstrated that considerable numbers of hepatic genes are also regulated by 5-hydroxymethylcytosine (5hmC) [23]. Therefore, the levels of 5hmC should be evaluated in our next studies.

Interestingly, *CYP1A2*, *CYP2C19*, *CYP2D6*, and *SULT1A1* tend to show inter-individual variations in activity, and this has become a major problem in clinical practice. Our data supported that these variations may result from the variable DNA methylation statuses of these DME genes. For example, DNA methylation status could explain the discrepancy in the relatively variable expression of *CYP2C19* among Caucasian populations in which there is a low frequency of mutant genotypes [4]. Although considerable inter-individual differences were observed in *CYP3A4* expression (CV 22.9 %; Fig. 2b), we did not detect a significant relationship between DNA methylation and mRNA expression. As reported by Kacevska et al. [24], DNA methylation at more 5′ distal regions (position −1547 or −10,762) may be associated with *CYP3A4* regulation. Alternatively, *PXR* gene methylation, but not *CYP3A4* gene methylation, may contribute to *CYP3A4* downregulation in colon cancer cells, as previously described by our laboratory [14].

We also found five DME genes (*CYP1B1*, *CYP8B1*, *GSTM2*, *GSTP1*, and *UGT3A2*) that were likely to be downregulated by DNA methylation. Further analyses, such as chromatin immunoprecipitation, will be required to establish functional evidence for this relationship. Interestingly, the methylation statuses of these five DME genes were highly stable in normal livers, suggesting that DNA methylation may play different roles in the regulation of DME genes between normal and cancerous livers. As expected, DNA methylation in the 5′-regulatory region of housekeeping and tumor suppressor genes was stable and restricted to low levels. In contrast, the methylation levels of these genes were relatively high within the exons. These results were supported by a previous study showing that DNA methylation plays critical roles as markers of exon-intron boundaries and transcriptional silencing [25, 26].

In this study, we demonstrated that DNA methylation could function as a guide for correct alternative splicing of the *UGT1A* gene in a tissue-specific manner. Despite the limited cases in our study, our findings were supported by recent genome-wide analyses showing that many genes are regulated by alternative splicing via DNA methylation [18, 25, 26]. Oda et al. also reported that tissue-specific *UGT1A10* expression is regulated by DNA methylation [27]. We found that the relatively low expression levels of DME genes in the fetal liver could be associated with the higher methylation levels observed in these genes. Thus, DNA methylation mapping may provide novel insights into the tissue-specific and age-dependent splicing switch of DME genes regulated by DNA methylation.

From these results, we propose that the roles of DME genes in the liver depend on the DNA methylation landscape (Fig. 7). Variable methylation of the DME gene observed in normal livers may be one of the mechanisms mediating inter-individual variation in drug metabolizing activity and drug efficacy if the methylation status is associated with mRNA expression. Therefore, the DNA methylation statuses of the seven HVM-type genes may be indicators for prediction of drug efficacy and safety. Although there were considerable number of DME genes that showed highly variable DNA methylation (maximum *β*_R_ > 0.296) without inverse correlation to gene expression, such DNA methylation was probably unrelated to gene expression and other mechanisms might be involved in the regulation of these DME genes. The cutoff value as maximum *β*_R_ > 0.296 may not be the best for categorizing HVM type, because maximum *β*_R_ can depend on the number of CpG sites examined for each gene. However, some DME genes such as *CYP1A2* are regulated by DNA methylation at exclusively confined CpG sites [28]. In order to detect any informative signals without averaging effect, therefore, we evaluated *β*_R_ value with single (maximum) CpG site rather than with the fraction of CpG sites. We also found five TSG-type genes that were thought to act as tumor suppressor genes because they exhibited DNA methylation features similar to those observed in the *BMP4* and *IGFBP3* genes. Therefore, these TSG-type genes may have novel functions related to gatekeeping or genome stability in tumor cells. On the other hand, some HKG-type genes were suggested to function as housekeeping genes. Based on this extremely stable DNA methylation status, we predicted that the expression of these HKG-type genes should be tightly regulated, which may explain why DME genes with stable DNA methylation had conserved CpG islands. Interestingly, DMEs with a small range of variation in DNA methylation were known to metabolize endogenous substrates rather than xenobiotics. In contrast, DMEs with highly variable DNA methylation (including *CYP1A2*, *CYP2C19*, and *CYP2D6*) catalyze the metabolism of many xenobiotics, including drugs used in the clinical setting. These types of DMEs may flexibly modify their DNA methylation and expression profiles to metabolize and excrete various xenobiotics. We hypothesize that DNA methylation may play different roles in the regulation of DME genes depending on context. For example, HKG-type genes are regulated by “rigid” DNA modifications to strictly retain the epigenome. On the other hand, HVM-type genes are regulated by “plastic” DNA modifications to flexibly rewrite the epigenome for adjustment to new environments. Thus, HVM-type genes tend to show higher inter-individual variations in DNA methylation status.

## Conclusions

We described the global DNA methylation landscape of DME genes in human tissues and demonstrated that a small but specific fraction of DME genes was regulated by DNA methylation. Variations in DNA methylation may result in inter-individual differences in the efficacies and toxicities of many drugs. DNA methylation of DME genes may represent landmarks of tissue-specific and age-dependent splicing switches and may be useful indicators for predicting the unknown functions, such as tumor-suppressor or housekeeper.

## Methods

### Cell culture and treatment

Human hepatoma HepG2, HuH7, and JHH1 cells were obtained from American Type Culture Collection (Manassas, VA, USA) and cultured in Dulbecco’s minimal essential medium (Invitrogen, Carlsbad, CA, USA) containing 10 % fetal bovine serum at 37 °C in a humidified atmosphere containing 5 % CO_2_. In order to reverse DNA methylation, the cells were treated with 0.5 or 5 μM 5-aza-2′-deoxycytidine (DAC; Sigma-Aldrich, St. Louis, MO, USA) for 72 h. After DAC treatment, the cells were treated with trichostatin A (TSA; Sigma-Aldrich) at 200 nM (HepG2 and HuH7 cells) or 20 nM (JHH1 cells) for 24 h.

### DNA and RNA samples

Genomic DNA was extracted from hepatoma cells using a standard proteinase K/sodium dodecyl sulfate and phenol/chloroform method. Total cellular RNA was isolated using an RNeasy Mini Kit (Qiagen, Hilden, Germany) according to the manufacturer’s instructions. Genomic DNA and total RNA of human adult liver tissues (NLA and NL2), fetal liver tissue (NLF), adult small intestinal tissue (NSI), and adult colonic tissue (NC) were obtained from commercially available products (Capital Biosciences, Gaithersburg, MD, USA and BioChain Institute, Newark, CA, USA). Because we had no information other than age, gender, and race (Chinese) for these samples, we did not submit our research proposal to an ethical committee.

### DNA methylation analysis

Genomic DNA from tissues and hepatoma cells was subjected to sodium bisulfite modification using an EpiTect Plus DNA Bisulfite Kit (Qiagen) and examined for DNA methylation using an Infinium HumanMethylation450 Bead Chip (Illumina, San Diego, CA, USA), which interrogates over 480,000 CpG sites in the genome [29]. The level of DNA methylation for each CpG site was reported as the *β* value, which ranged from 0 (fully unmethylated) to 1 (fully methylated). Data have been deposited in the GEO repository with accession numbers GSE67477 and GSE67484 (Additional file 10: Table S2). DNA methylation data of 55 *CYP* genes and 62 phase II DME genes, including 17 glutathione *S*-transferase (*GST*), 10 *N*-acetyltransferase (*NAT*), 13 sulfotransferase (*SULT*), and 22 *UGT* genes, were selected and used for subsequent analyses. For the control, four housekeeping genes (*ACTB*, *B2M*, *GAPDH*, *TBP*), two tumor suppressor genes (*BMP4* and *IGFBP3*), and two DNA repair genes (*MLH1* and *MGMT*) were also examined. The results were visualized in heat maps using Cluster 3.0 software (http://bonsai.hgc.jp/~mdehoon/software/cluster/software.htm#ctv). The DNA methylation status of candidate DME genes was validated by COBRA method [30]. Briefly, after polymerase chain reaction (PCR) using a TaKaRa EpiTaq HS kit (Takara Bio Inc., Shiga, Japan), the resulting products were digested with an appropriate restriction enzyme, such as HpyCH4IV, TaqI, or BstUI (New England Biolabs, Ipswich, MA, USA). The digested products were electrophoresed on 2 % agarose gels followed and visualized by ethidium bromide staining. Primers used are listed in Additional file 11: Table S3.

### mRNA expression analysis

First-strand cDNA was synthesized from total RNA samples using a Transcriptor First Strand cDNA Synthesis Kit (Roche Diagnostics, Mannheim, Germany) according to the manufacturer’s protocol. The mRNA expression profiles were examined using a SurePrint G3 Human Gene Expression 8 × 60K v2 microarray (Agilent Technologies, Palo Alto, CA, USA). The signal intensity for each probe was normalized by the 75th percentile. The data have been deposited in the GEO repository with the accession number GSE67318 (Additional file 10: Table S2). The mRNA expression data for the *CYP* gene, phase II DME genes, and control genes were selected and used for subsequent analyses. The level of each transcript was validated by quantitative real-time PCR analysis using a FirstStart Universal SYBR Green Master (ROX) kit (Roche Diagnostics). Transcript levels of individual *UGT1A* isoforms and *ACTB* were evaluated by TaqMan Gene Expression Assays (Life Technologies, Gaithersburg, MD, USA). All primer sets used were described in Additional file 11: Table S3. Each real-time PCR analysis was performed in triplicate.

### GEO datasets used for integrative analysis

DNA methylation datasets of liver tissues derived from 18 healthy German individuals were found in GEO records (GSE48325) [21]. These data were obtained in another study using the same HumanMethylation450 platform (GPL13534) and could be combined with our data for two adult liver tissues (NLA and NL2). Moreover, transcript datasets were also found for 10 of the 18 liver tissues in GEO records (GSE48452) and used for correlation analysis between DNA methylation and mRNA expression.

### DNA methylation mapping

DNA methylation mapping was carried out for two purposes. First, in order to estimate the variable DNA methylation statuses of the 20 adult liver tissues, the *β* value of each CpG site was expressed as a box-and-whisker plot. Second, we aimed to identify informative CpG sites in which DNA methylation levels were different between tissues or cell lines. We constructed line graphs connecting each CpG site arranged in the 5′ to 3′ direction according to the relative location and distance of each CpG site.

### Statistical analysis

Correlations between DNA methylation levels of each CpG site were examined for all CpG sites in the 5′ regulatory region by Spearman’s rank correlation coefficient in 10 liver tissues. In the DNA methylation mapping of the *UGT1A* isoforms, Wilcoxon signed rank test was used to compare median of 18 individual *β*_R_ values of normal adult liver and one normal small intestine (NSI) or one normal fetal liver (NLF). These analyses were performed using GraphPad Prism 5 software (GraphPad Software, La Jolla, CA, USA), and differences or correlations with *p* values of less than 0.05 were considered significant.

## Availability of supporting data

1. GSE67477 (DNA methylation)
2. GSE67484 (DNA methylation)
3. GSE67318 (mRNA expression)

This series is linked to GSE67485.

## Additional files

Additional file 1: Figure S1. Results of methylation mapping of 20 liver tissues. In each panel, the horizontal axis indicates the positions of CpG sites arranged in the 5′ to 3′ direction. The 5′ regulatory region (TSS1500, TSS200, or 5′UTR) is underlined. The vertical axis indicates the *β* values of individual CpG sites with the variations expressed as box-and-whisker plots. The maximum *β*_R_ value within the 5′ regulatory region is indicated by a circle for each gene. (PDF 377 KB) Additional file 2: Figure S2. Distribution of the mRNA expression levels and CV values of DME and control genes. (A) The horizontal axis of the histogram represents the logarithm of the mean value of the transcript (normalized values by the 75th percentile) for 10 liver specimens. (B) The horizontal axis of the histogram represents the CV (%) of the transcript. (PDF 33.6 KB) Additional file 3: Figure S3. Representative results of correlation analysis. Significant inverse correlations between DNA methylation levels and mRNA expression levels were detected for the other five DME genes (*CYP1A2*, *CYP2D6*, *GSTM5*, *GSTT1*, and *SULT1A1*; *p* < 0.05, Spearman’s rank correlation test). The mRNA expression level, shown as the vertical axis, was normalized to the level of *ACTB* mRNA (set as 100 %). CpG sites with significant correlation are indicated by asterisks in the methylation map for each gene. CpG sites showing *β*_R_ values of more than 0.296 are indicated by circles. (PDF 54.3 KB) Additional file 4: Figure S4. DNA methylation profiles of DME genes in three hepatoma cell lines. DME genes with hypermethylated or hypomethylated CpG sites are listed and shown by closed and shaded boxes, respectively. The number of CpG sites showing hypermethylation or hypomethylation is also indicated in each box. (PDF 31.9 KB) Additional file 5: Figure S5. Changes in the mRNA expression levels of DME genes in three hepatoma cell lines after DAC treatment. DME genes showing upregulation in at least one hepatoma cell line (greater that twofold change) following DAC treatment are listed. The fold change is indicated in each box, and genes with fold changes of more than two are indicated by closed boxes. The six DME genes exhibiting correlations between mRNA expression and DNA methylation status are shown in highlighted boxes. (PDF 37.9 KB) Additional file 6: Figure S6. DNA methylation mapping of *BMP4* and *IGFBP3* tumor suppressor genes in the three hepatoma cell lines. In each panel, open circles located on the horizontal axis indicate the positions of CpG sites arranged in the 5′ to 3′ direction according to the relative distance of each CpG site. Exons are indicated by green boxes, and translated regions are shown in dark green. The fold change in mRNA expression is shown on the right side of the panel. Following DAC treatment, the *BMP4* and *IGFBP3* genes were exclusively upregulated in JHH1 (7.0 fold) and HepG2 (8.8 fold) cells, respectively, and hypermethylation was also detected in methylation mapping for these two cell lines, with *β* values of more than 0.5. (PDF 167 KB) Additional file 7: Figure S7. Expression levels of *UGT1A* transcripts in different tissues. The vertical axis indicates the mRNA expression levels of *UGT1A* isoforms normalized to the level of *UGT1A1* expression in adult liver (NL2; set as 100 %). Each column represents the mean ± SD (*n* = 3). (PDF 35.5 KB) Additional file 8: Figure S8. DNA methylation mapping on the *UGT1A* locus. The vertical axis of the upper panel shows the level of DNA methylation on the *UGT1A* locus for different tissues. Open circles on the horizontal axis indicate the positions of CpG sites corresponding to the *UGT1A* gene structure shown in the lower panel. The *UGT1A* isoforms are transcribed by an exon sharing mechanism in which the transcripts of the individual first exon cassette are spliced to exons 2–5. The CpG sites boxed in red correspond to the first exon of each isoform. (PDF 179 KB) Additional file 9: Table S1. Levels of DNA methylation around the first exons of the *UGT1A* isoforms. The nine *UGT1A* isoforms were classified into two groups (hepatic and intestinal types). The levels of DNA methylation at individual CpG sites of the first exons were expressed as *β* values and separately compared between 18 adult livers and one normal small intestine (NSI) or one normal fetal liver (NLF). The *β* values colored in red were statistically higher than the median of *β* values of normal adult livers. The *β* values colored in blue were statistically lower than the median of *β* values of normal adult livers. The CpG sites proximal to TSS200 region (except TSS1500 region) were indicated by bold frame. (XLSX 15.2 KB) Additional file 10: Table S2. Datasets deposited in the GEO repository. (DOC 46.5 KB) Additional file 11: Table S3. Primers used for mRNA expression and DNA methylation analyses. (DOC 44.5 KB)

## Abbreviations

ACTB: actin, beta
B2M: β2 microglobulin
BMP4: bone morphogenetic protein 4
COBRA: combined bisulfite restriction analysis
CYP: cytochrome P450
DAC: 5-aza-2′-deoxycytidine
DME: drug metabolizing enzyme
GAPDH: glyceraldehyde-3-phosphate dehydrogenase
GEO: Gene Expression Omnibus
GST: glutathione *S*-transferase
IGFBP3: insulin-like growth factor binding protein 3
MGMT: *O*-6-methylguanine-DNA methyltransferase
MLH1: MutL homologue 1
NAT: *N*-acetyltransferase
SULT: sulfotransferase
TBP: TATA box-binding protein
TSA: trichostatin A
UGT: UDP-glucuronosyltransferase
UTR: untranslated region
