# Supplementary material for: Analysis of DNA methylation landscape reveals the roles of DNA methylation in the regulation of drug metabolizing enzymes
Source: Clin Epigenetics. 2015 Sep 28;7:105. doi: 10.1186/s13148-015-0136-7 (PMC4587720; doi:10.1186/s13148-015-0136-7)
Supplement: Additional file 1: Figure S1. — Results of methylation mapping of 20 liver tissues. In each panel, the horizontal axis indicates the positions of CpG sites arranged in the 5′ to 3′ direction. The 5′ regulatory region (TSS1500, TSS200, or 5′UTR) is underlined. The vertical axis indicates the β values of individual CpG sites with the variations expressed as box-and-whisker plots. The maximum β R value within the 5′ regulatory region is indicated by a circle for each gene. (PDF 377 KB) [file 13148_2015_136_MOESM1_ESM.pdf]

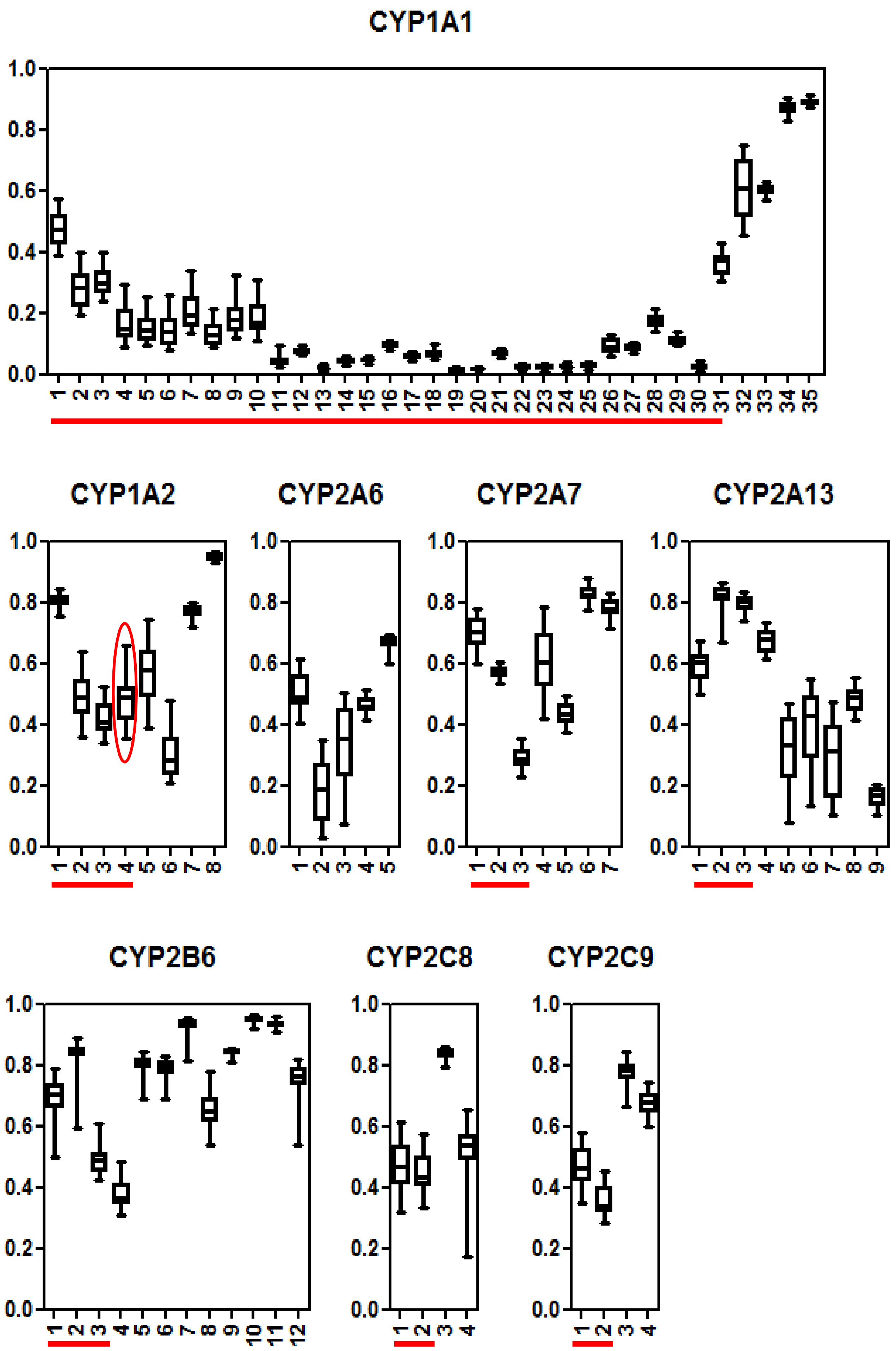
CYP1A1
0.0
0.2
0.4
0.6
0.8
1.0
1 2 3 4 5 6 7 8 9 10 11 12 13 14 15 16 17 18 19 20 21 22 23 24 25 26 27 28 29 30 31 32 33 34 35
CYP1A2
1 2 3 4 5 6 7 8
CYP2A6
1 2 3 4 5
CYP2A7
1 2 3 4 5 6 7
CYP2A13
1 2 3 4 5 6 7 8 9
CYP2B6
1 2 3 4 5 6 7 8 9 10 11 12
CYP2C8
1 2 3 4
CYP2C9
1 2 3 4

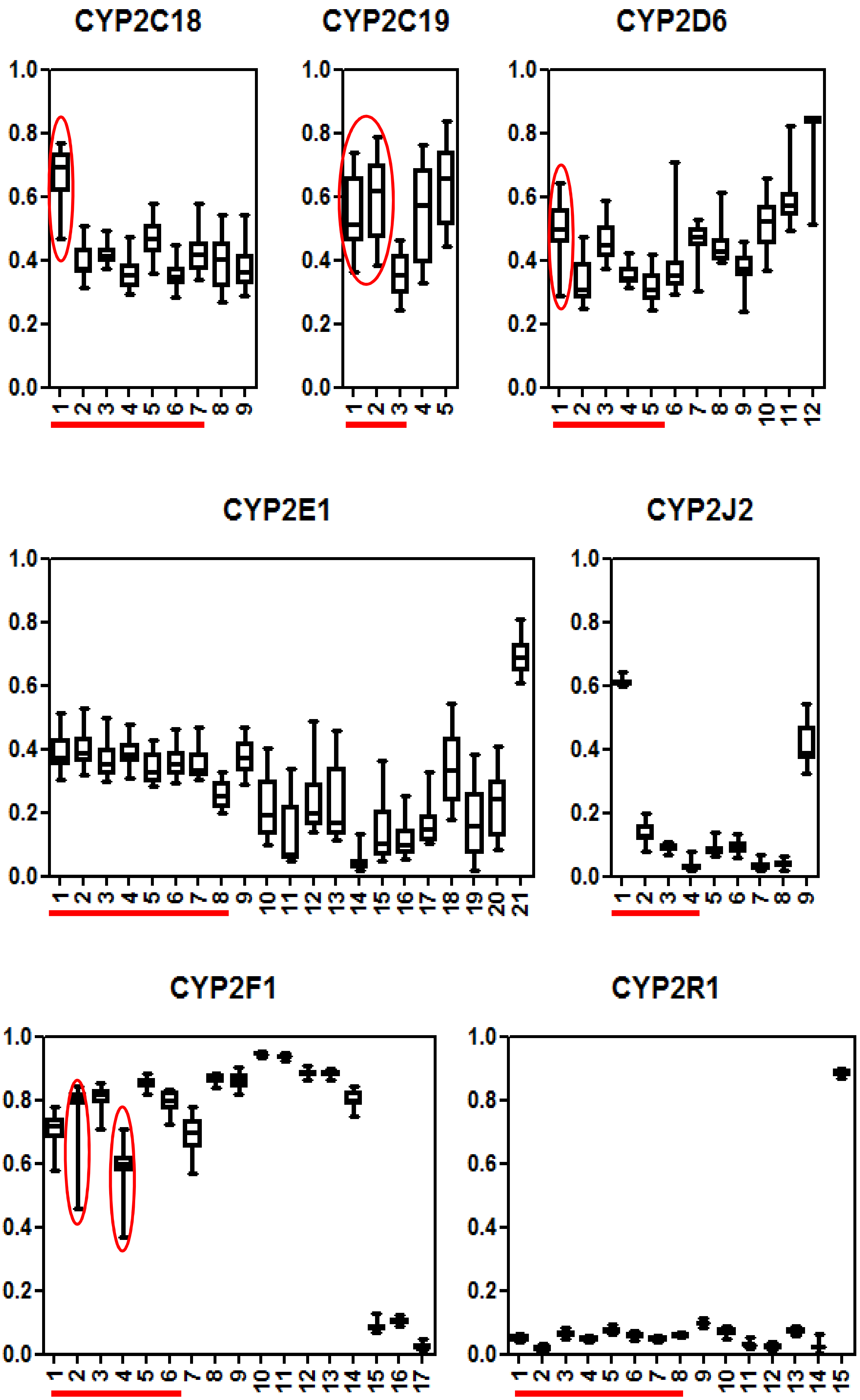
CYP2C18
CYP2C19
CYP2D6
CYP2E1
CYP2J2
CYP2F1
CYP2R1
1.0
0.8
0.6
0.4
0.2
0.0

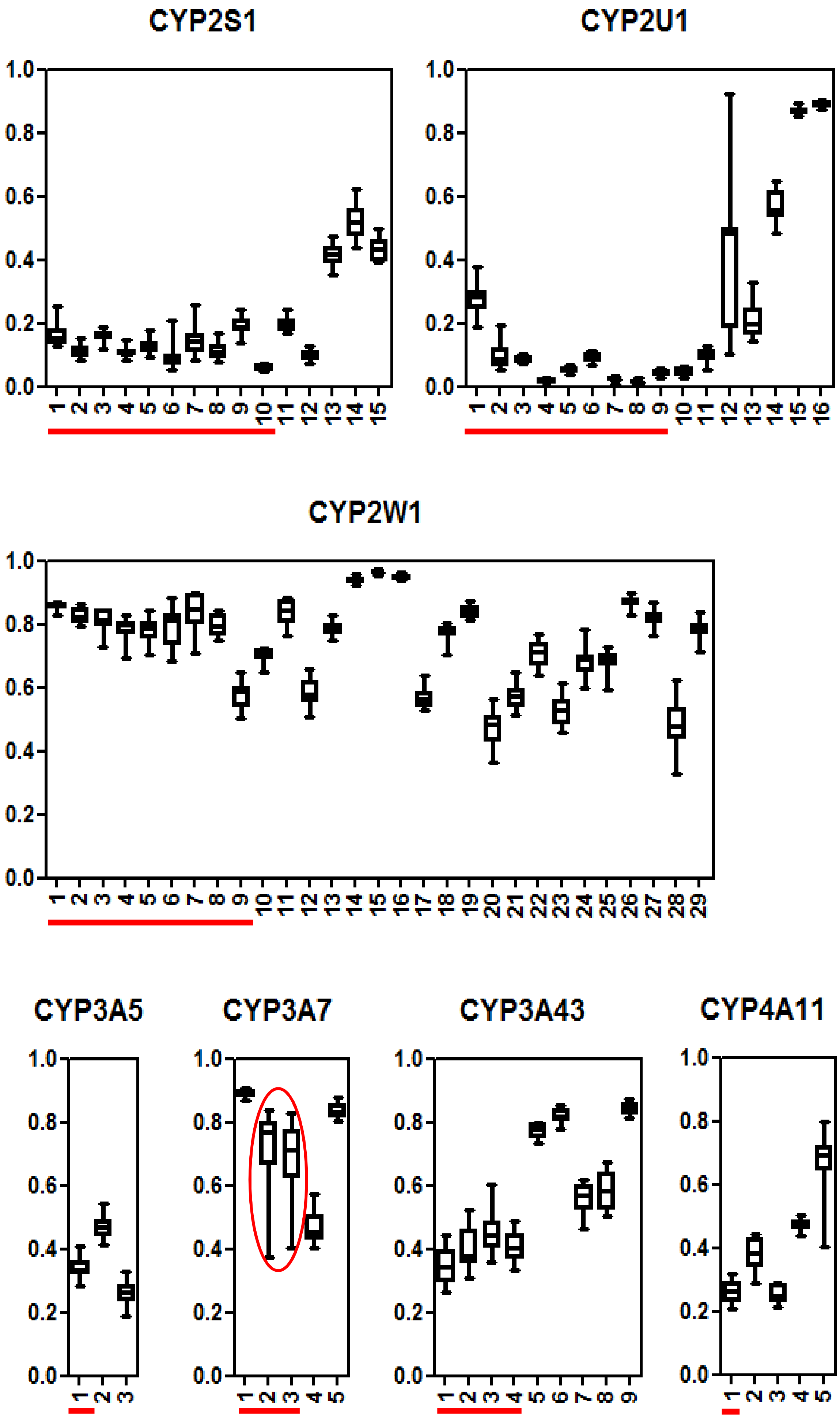
CYP2S1
CYP2U1
CYP2W1
CYP3A5
CYP3A7
CYP3A43
CYP4A11
1.0
0.8
0.6
0.4
0.2
0.0

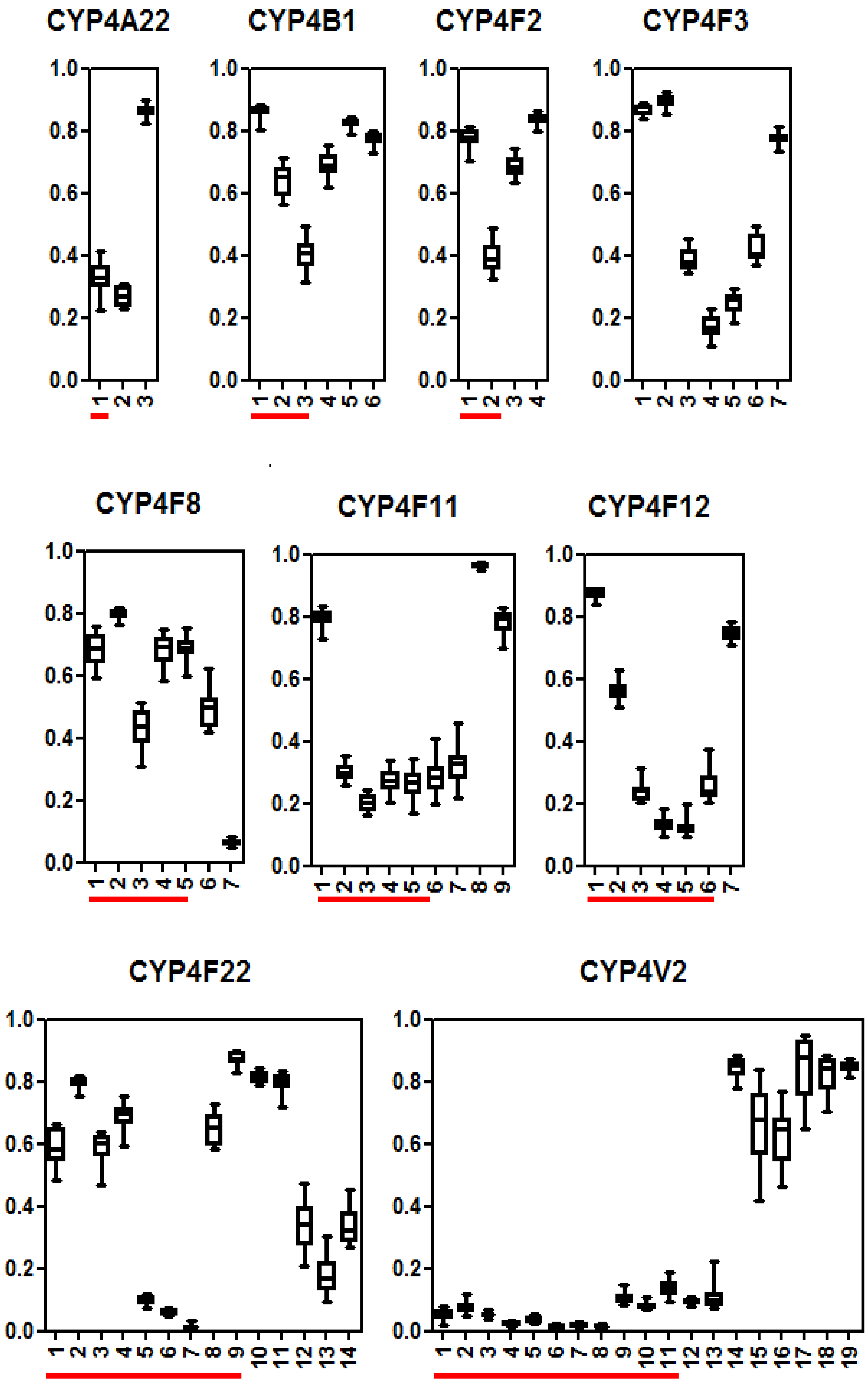
CYP4A22
CYP4B1
CYP4F2
CYP4F3
CYP4F8
CYP4F11
CYP4F12
CYP4F22
CYP4V2

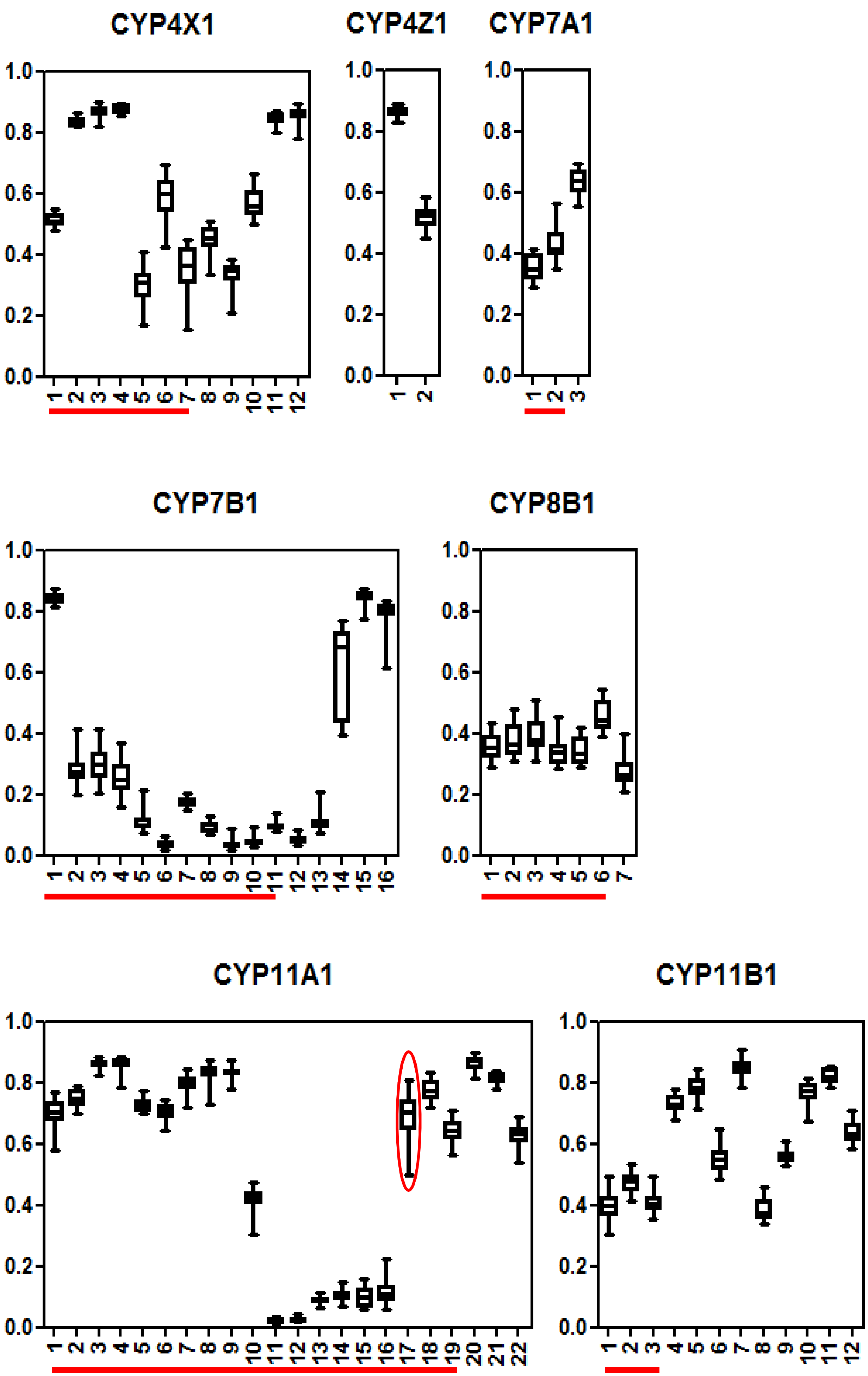
CYP4X1
CYP4Z1
CYP7A1
CYP7B1
CYP8B1
CYP11A1
CYP11B1
0.0
0.2
0.4
0.6
0.8
1.0

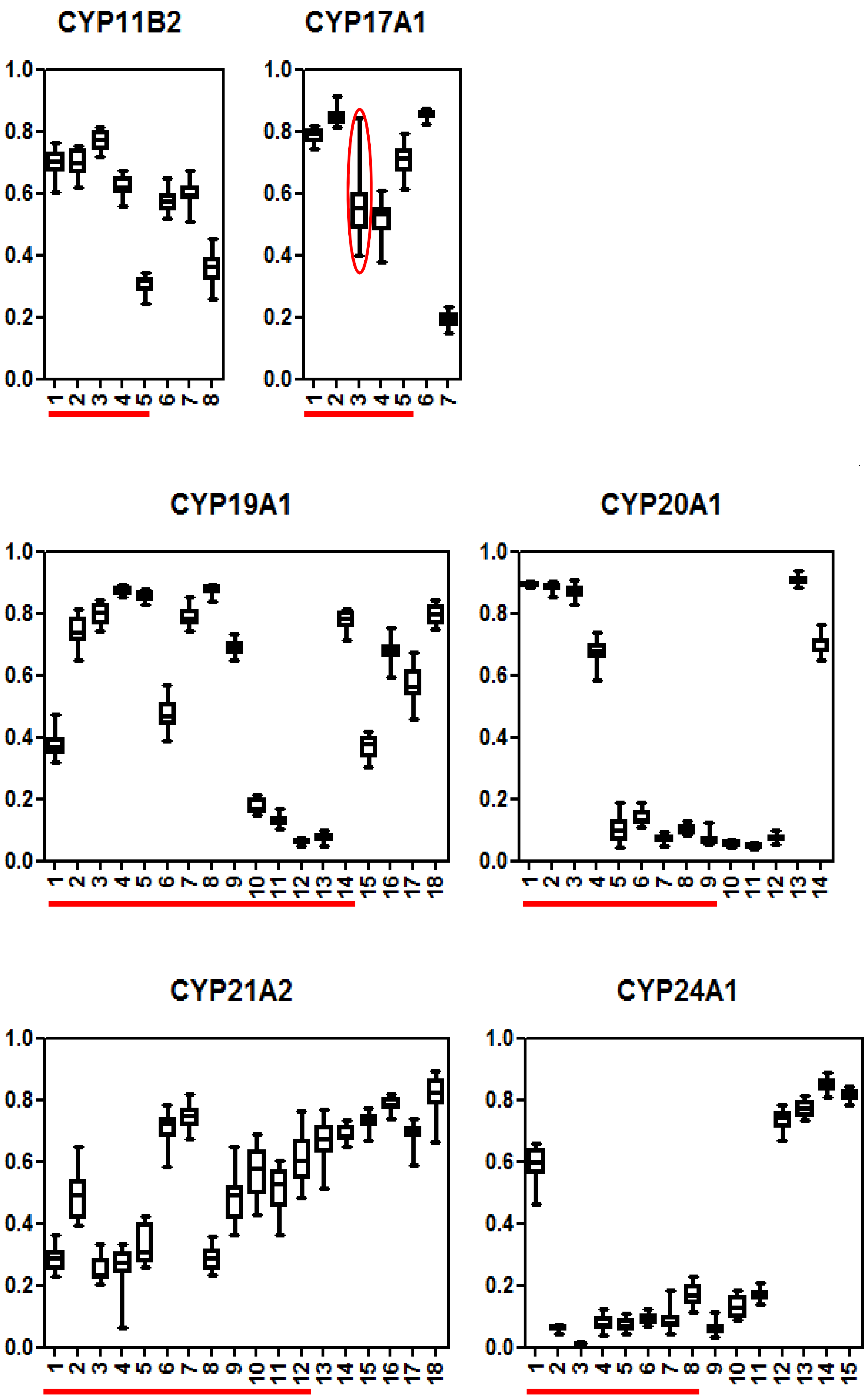
CYP11B2
CYP17A1
CYP19A1
CYP20A1
CYP21A2
CYP24A1
1.0
0.8
0.6
0.4
0.2
0.0

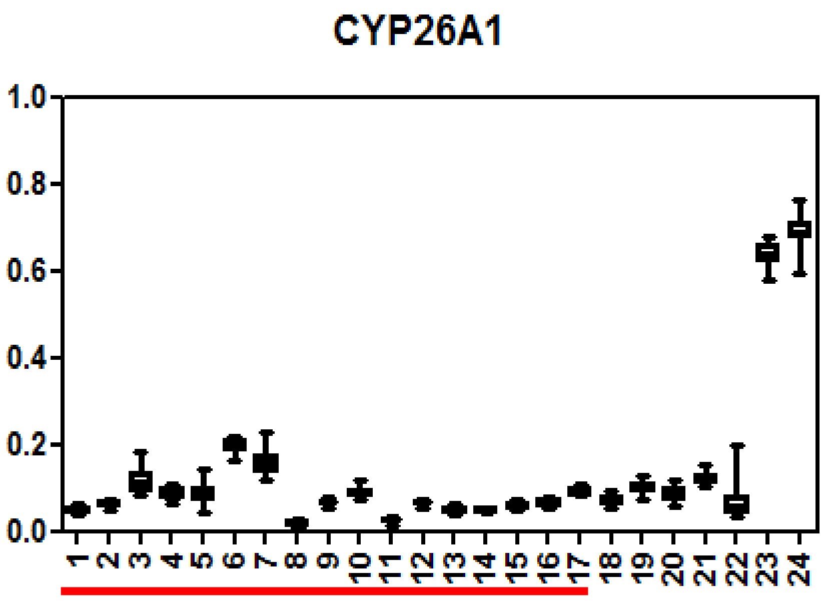
CYP26A1
1.0
0.8
0.6
0.4
0.2
0.0
1 2 3 4 5 6 7 8 9 10 11 12 13 14 15 16 17 18 19 20 21 22 23 24

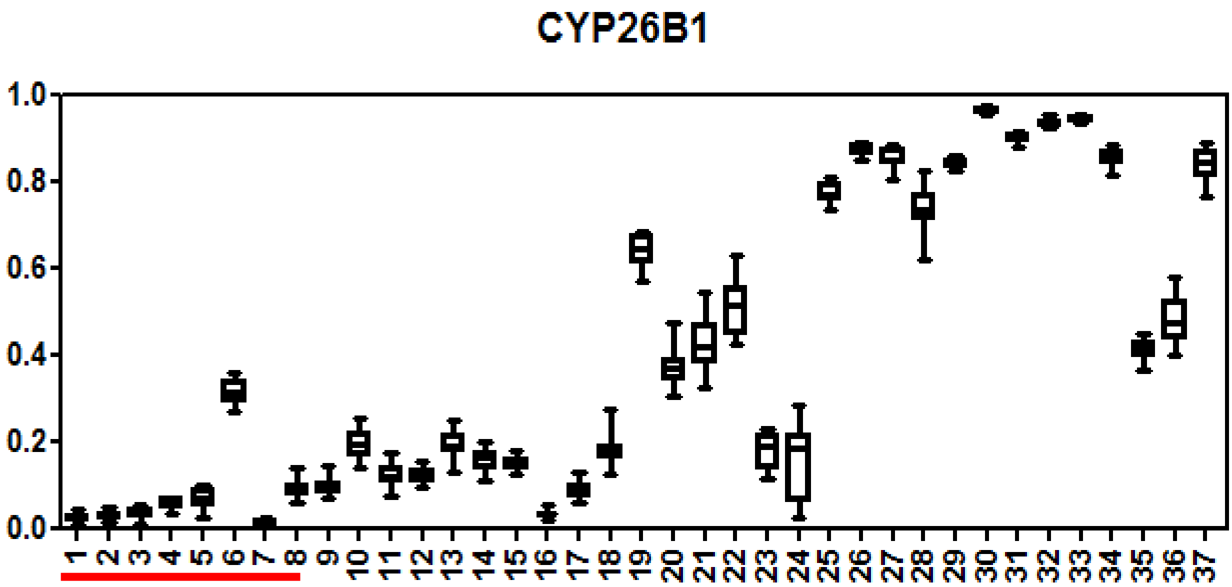
CYP26B1
1.0
0.8
0.6
0.4
0.2
0.0
1 2 3 4 5 6 7 8 9 10 11 12 13 14 15 16 17 18 19 20 21 22 23 24 25 26 27 28 29 30 31 32 33 34 35 36 37

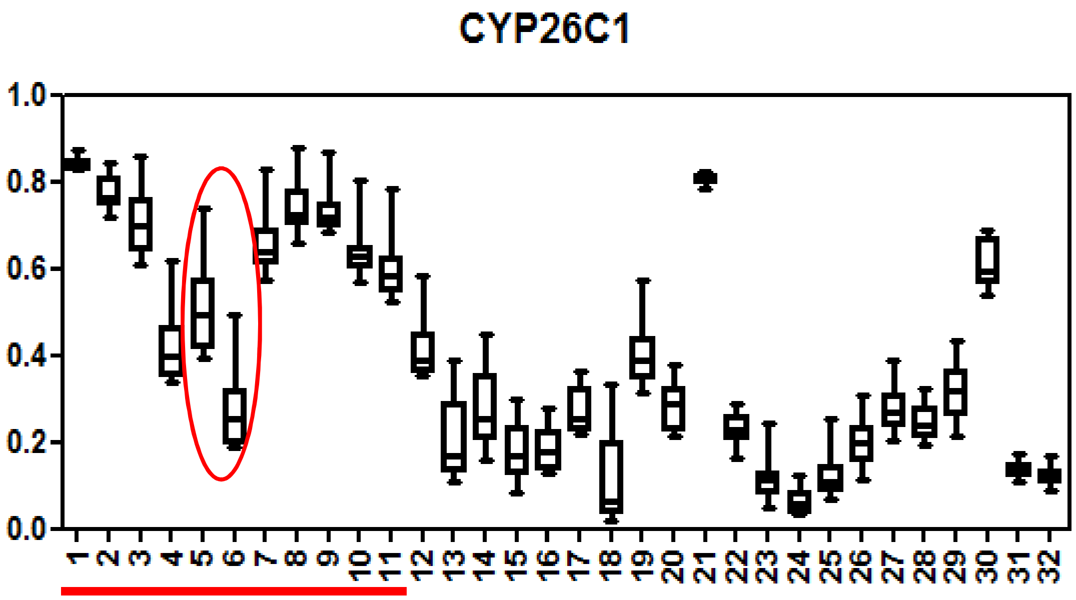
CYP26C1
1.0
0.8
0.6
0.4
0.2
0.0
1 2 3 4 5 6 7 8 9 10 11 12 13 14 15 16 17 18 19 20 21 22 23 24 25 26 27 28 29 30 31 32

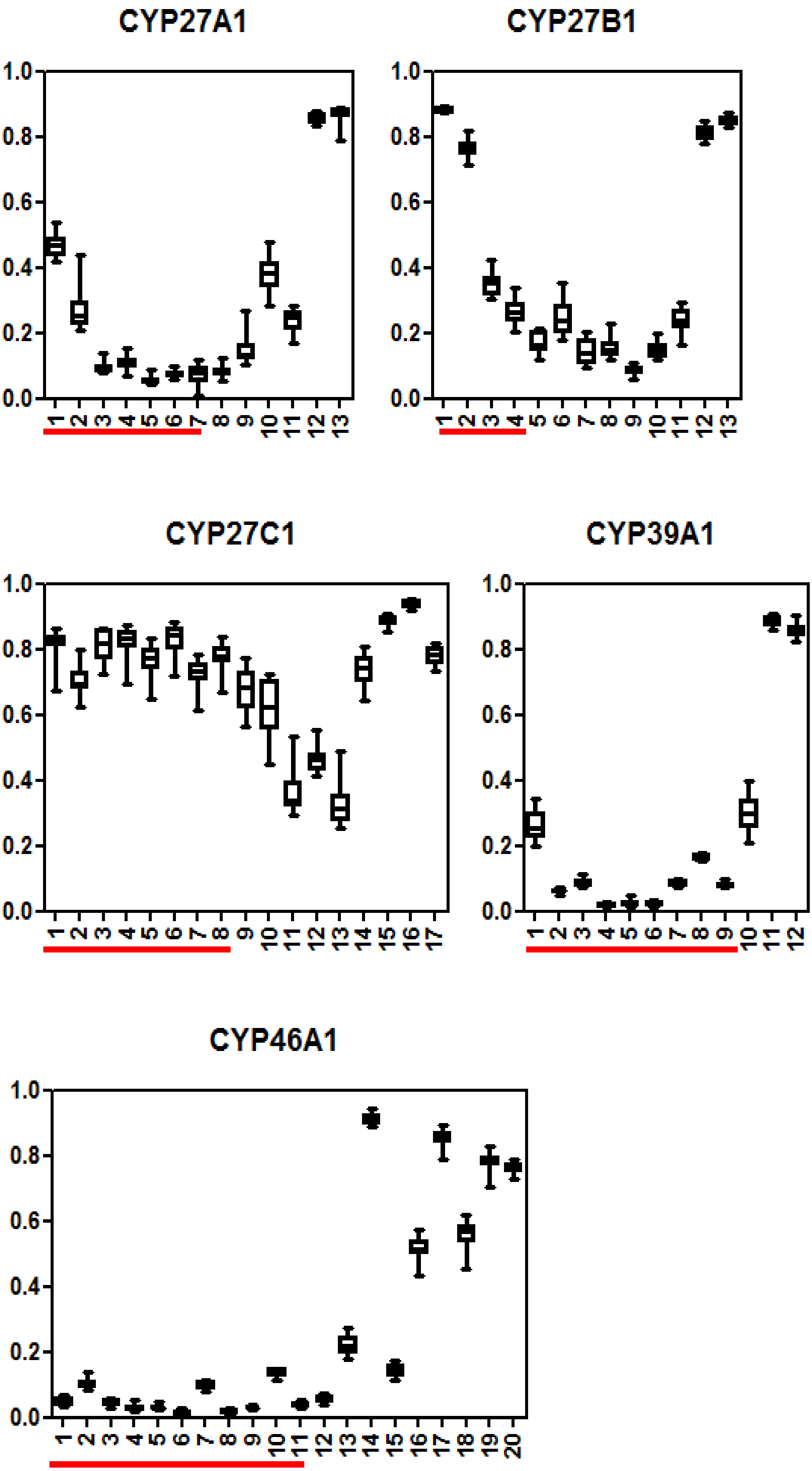
CYP27A1
CYP27B1
CYP27C1
CYP39A1
CYP46A1
1.0
0.8
0.6
0.4
0.2
0.0

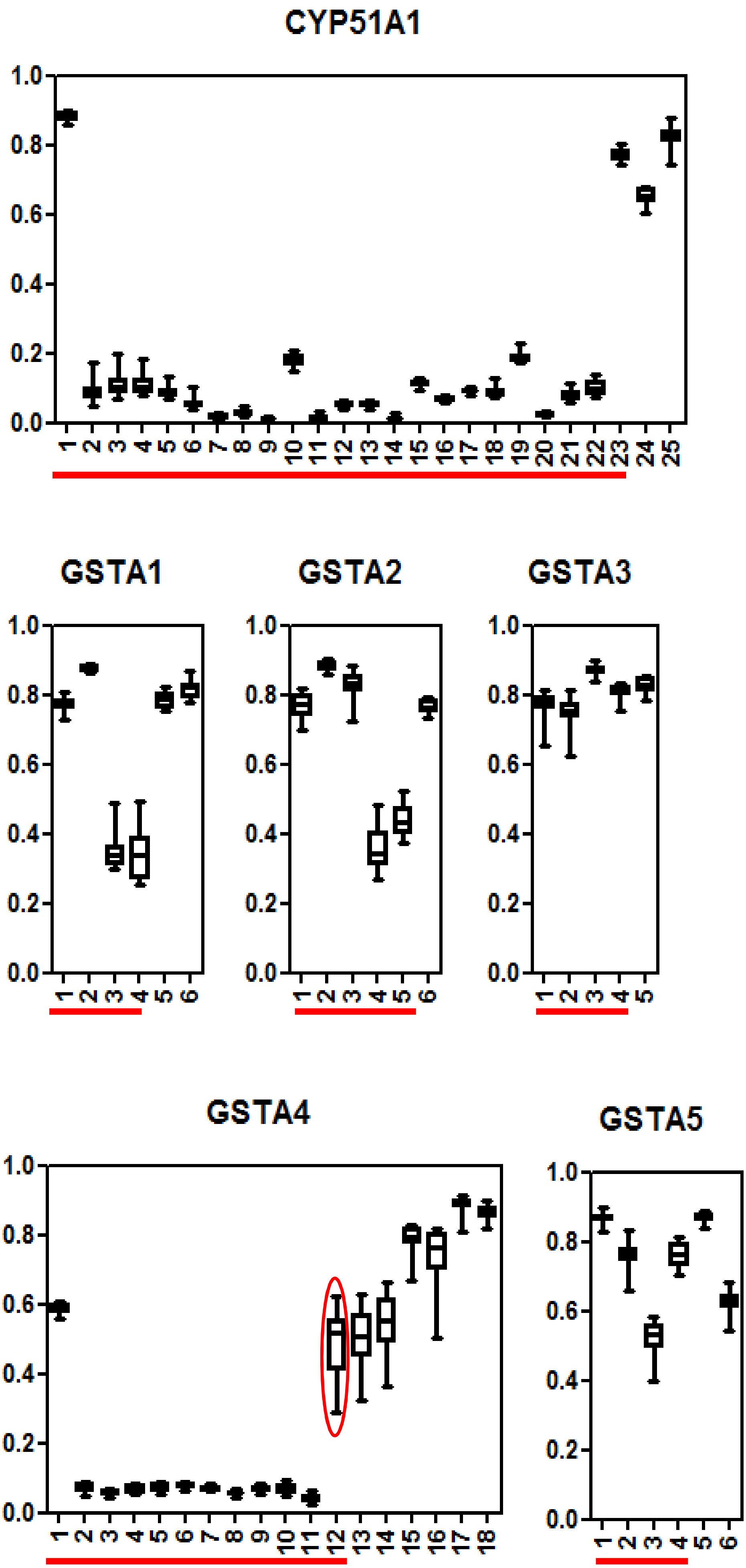

CYP51A1
1.0
0.8
0.6
0.4
0.2
0.0
1 2 3 4 5 6 7 8 9 10 11 12 13 14 15 16 17 18 19 20 21 22 23 24 25
GSTA1
GSTA2
GSTA3
1 2 3 4 5 6
1 2 3 4 5 6
1 2 3 4 5
GSTA4
GSTA5
1 2 3 4 5 6 7 8 9 10 11 12 13 14 15 16 17 18
1 2 3 4 5 6

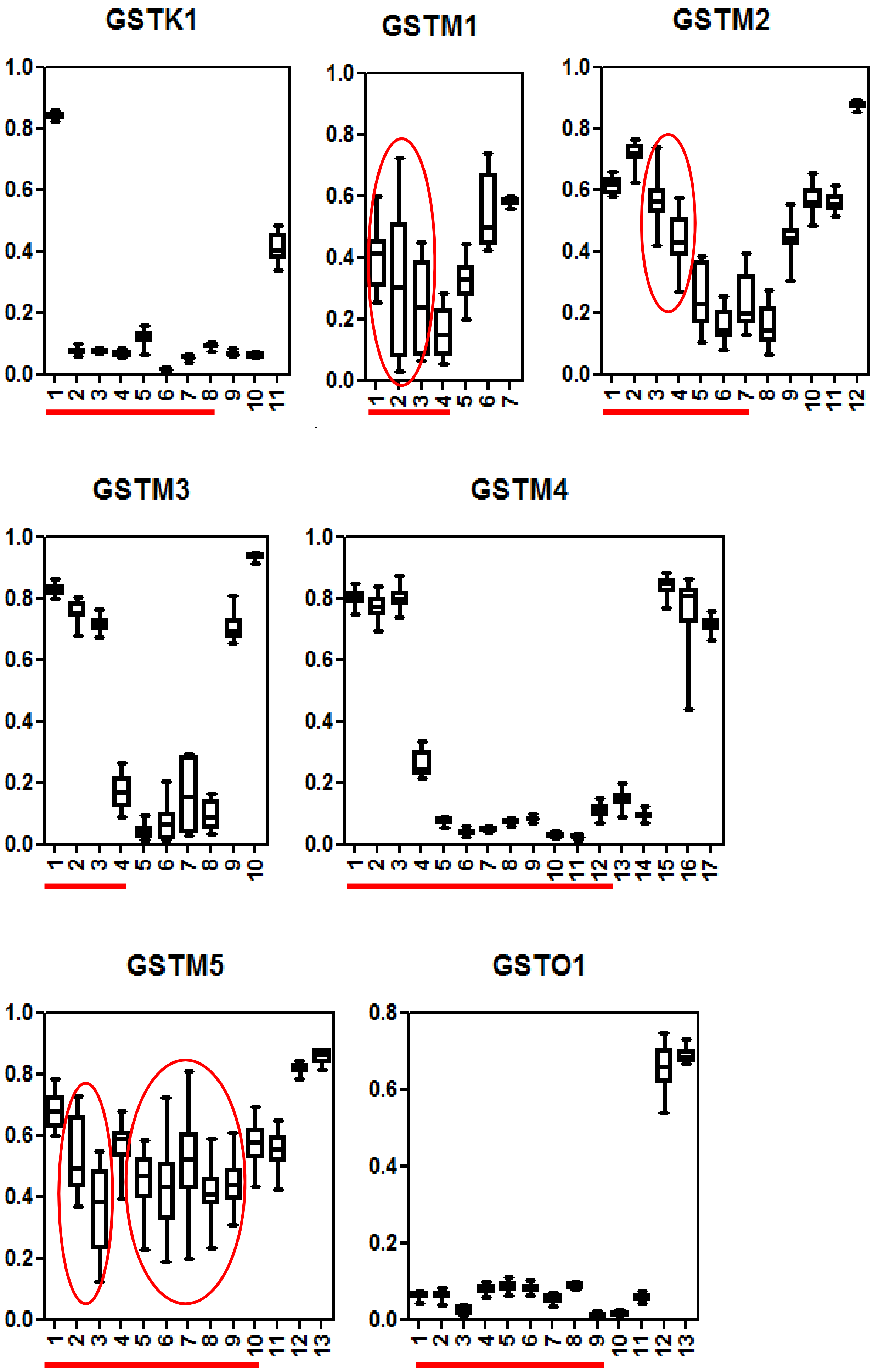

GSTK1
GSTM1
GSTM2
GSTM3
GSTM4
GSTM5
GSTO1

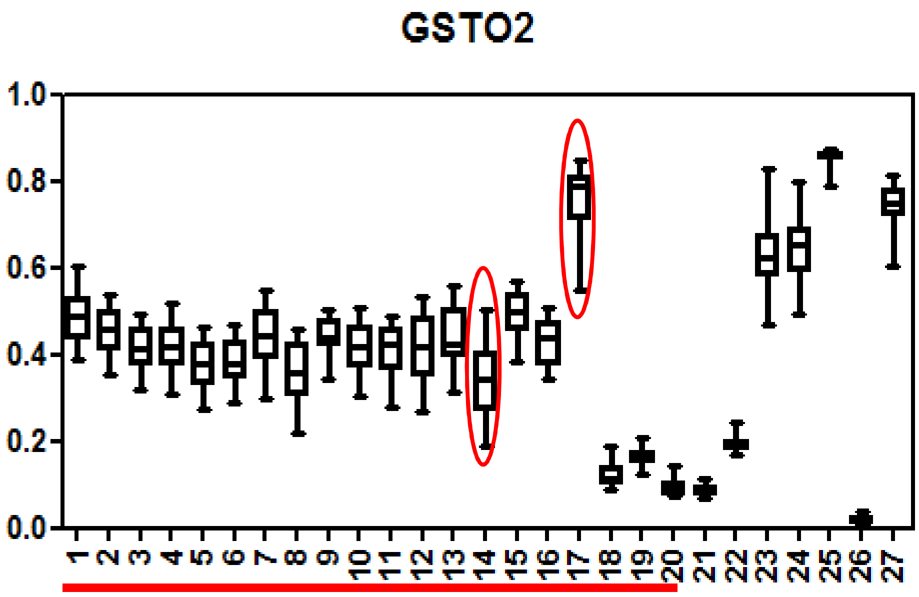
GSTO2
1.0
0.8
0.6
0.4
0.2
0.0
1 2 3 4 5 6 7 8 9 10 11 12 13 14 15 16 17 18 19 20 21 22 23 24 25 26 27

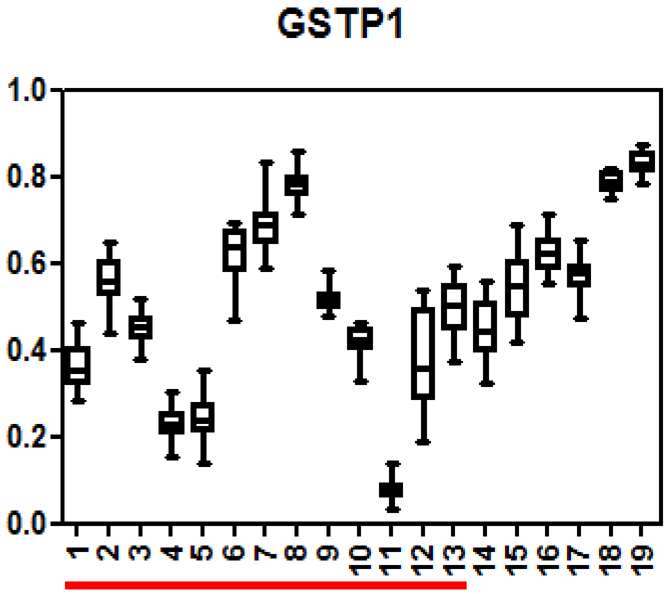
GSTP1
1.0
0.8
0.6
0.4
0.2
0.0
1 2 3 4 5 6 7 8 9 10 11 12 13 14 15 16 17 18 19

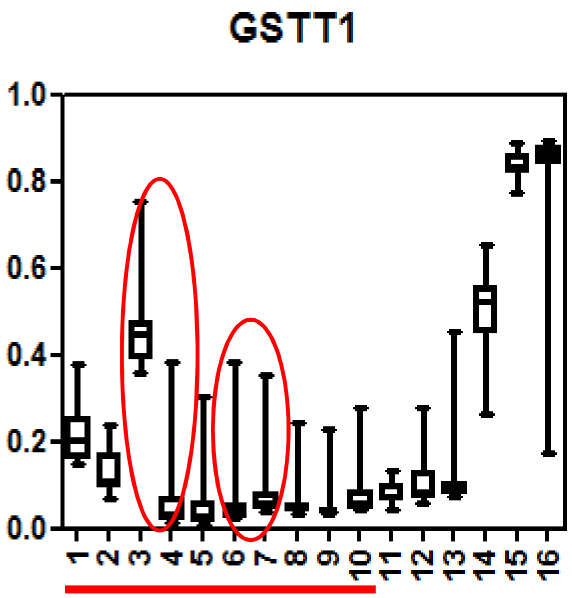
GSTT1
1.0
0.8
0.6
0.4
0.2
0.0
1 2 3 4 5 6 7 8 9 10 11 12 13 14 15 16

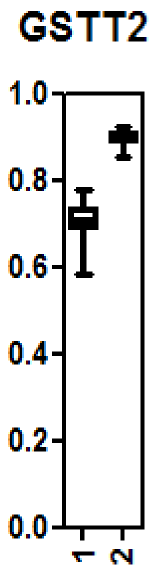
GSTT2
1.0
0.8
0.6
0.4
0.2
0.0
1 2

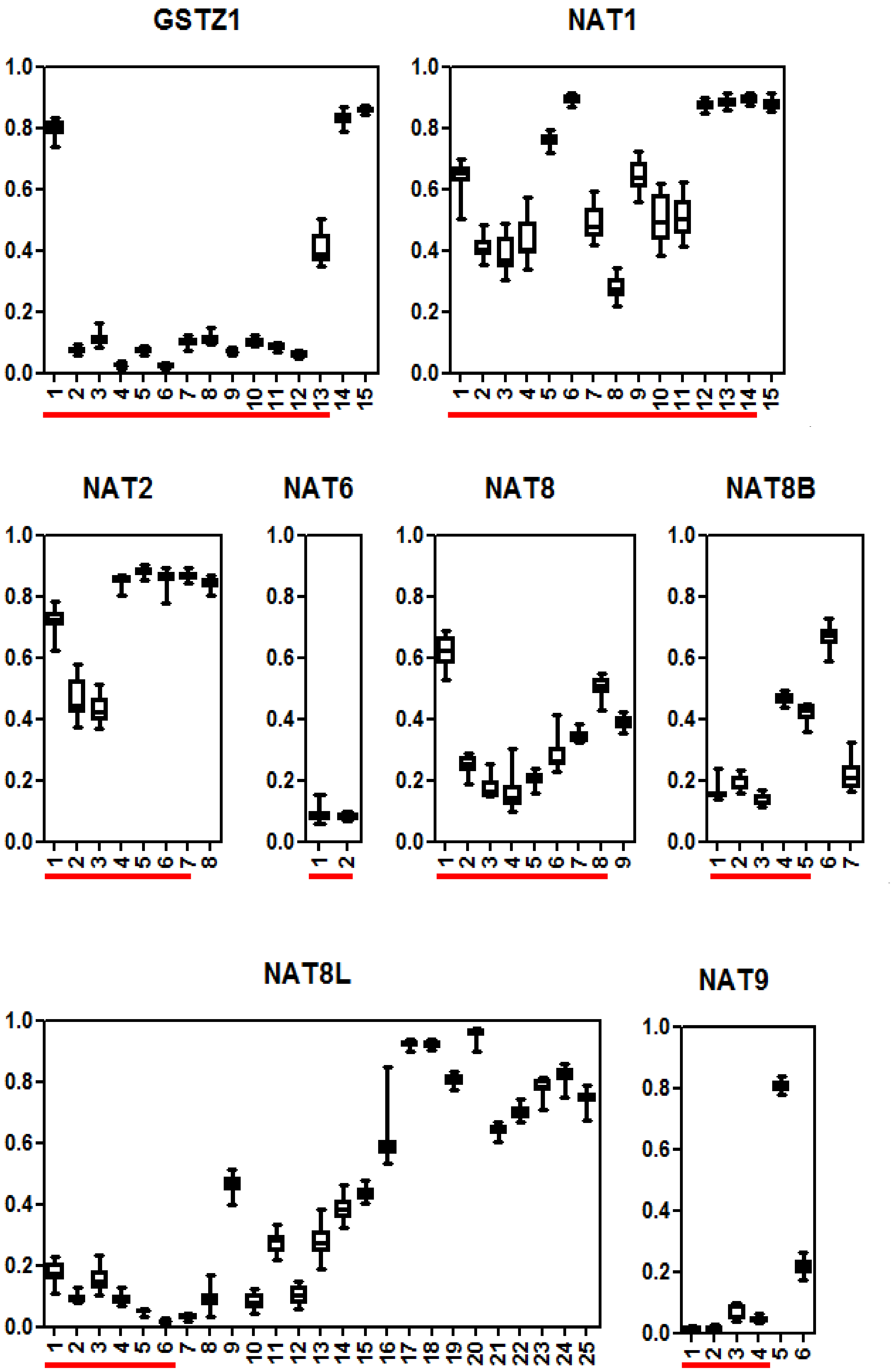
GSTZ1
NAT1
NAT2
NAT6
NAT8
NAT8B
NAT8L
NAT9
0.0
0.2
0.4
0.6
0.8
1.0

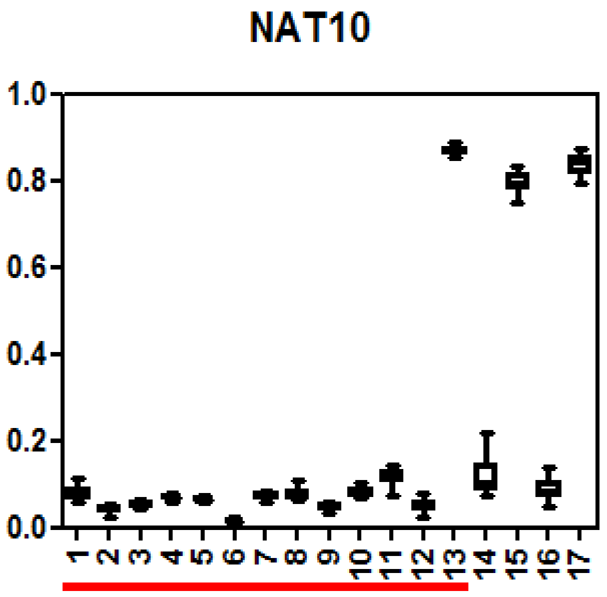
NAT10
1.0
0.8
0.6
0.4
0.2
0.0
1 2 3 4 5 6 7 8 9 10 11 12 13 14 15 16 17

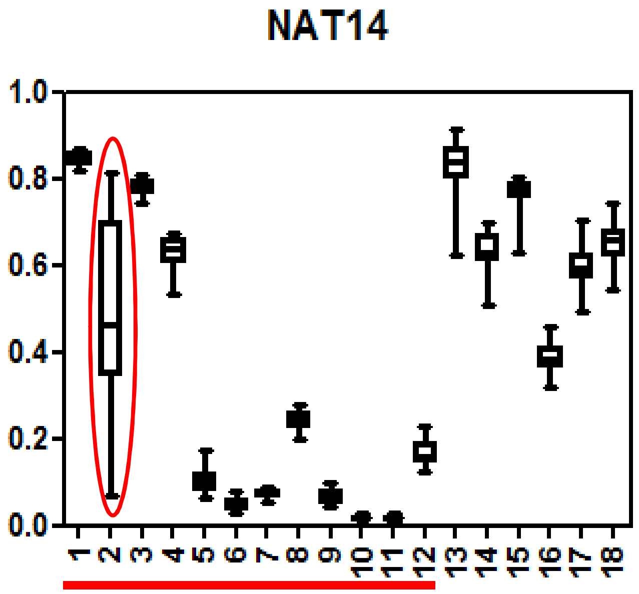
NAT14
1.0
0.8
0.6
0.4
0.2
0.0
1 2 3 4 5 6 7 8 9 10 11 12 13 14 15 16 17 18

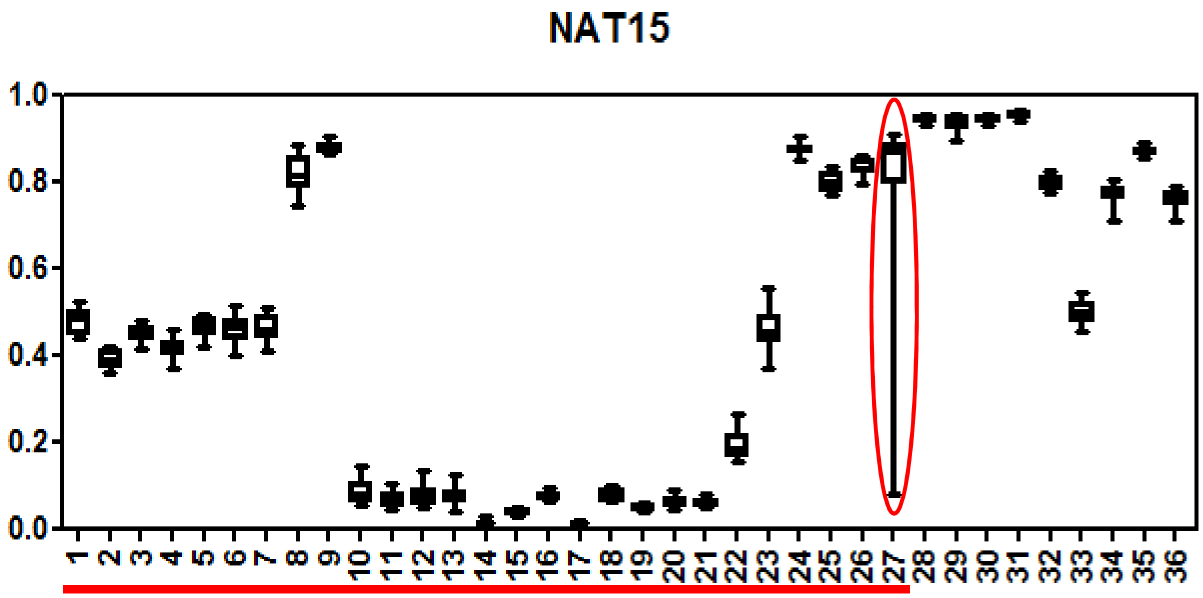
NAT15
1.0
0.8
0.6
0.4
0.2
0.0
1 2 3 4 5 6 7 8 9 10 11 12 13 14 15 16 17 18 19 20 21 22 23 24 25 26 27 28 29 30 31 32 33 34 35 36

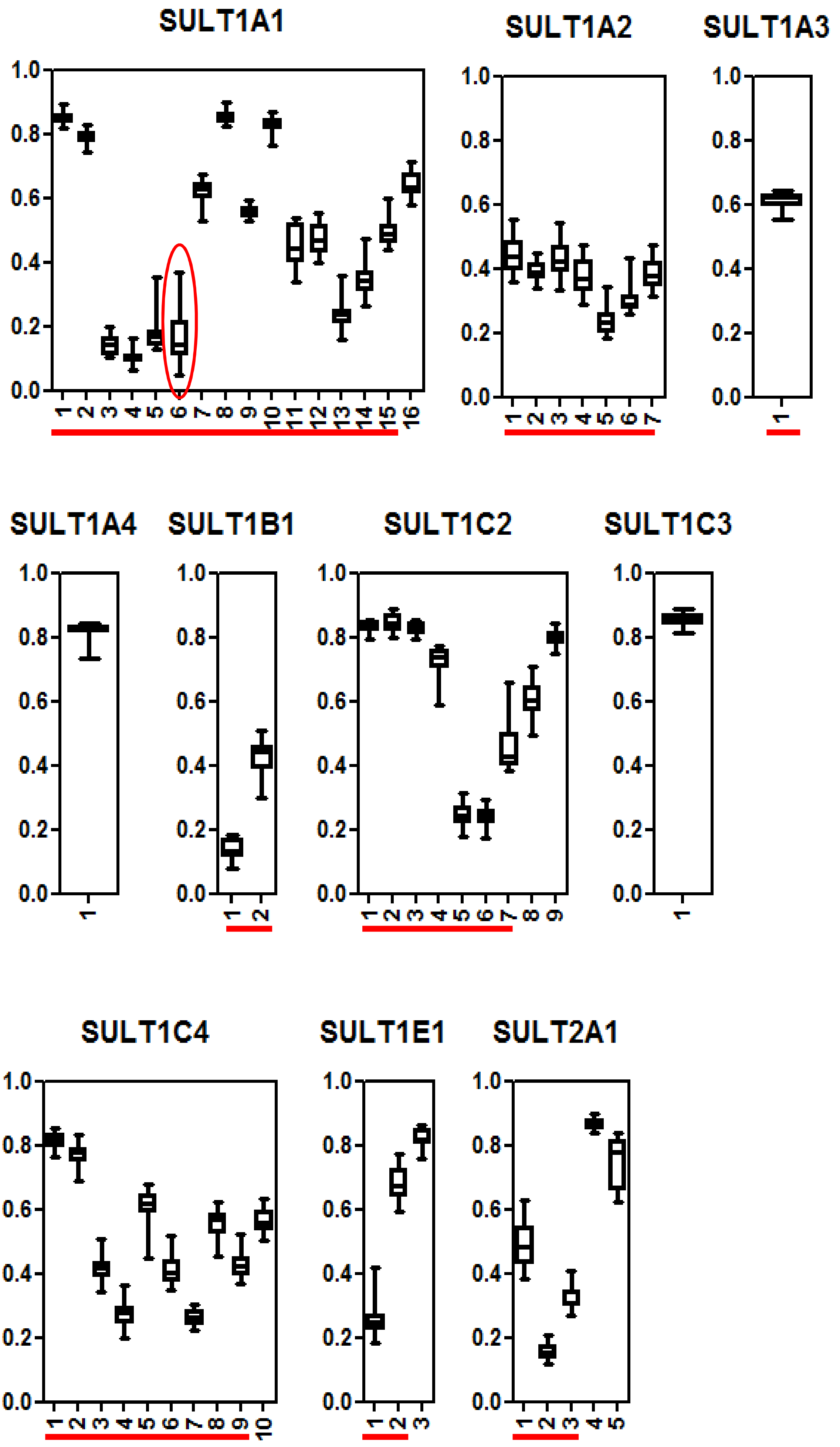
SULT1A1
SULT1A2
SULT1A3
SULT1A4
SULT1B1
SULT1C2
SULT1C3
SULT1C4
SULT1E1
SULT2A1

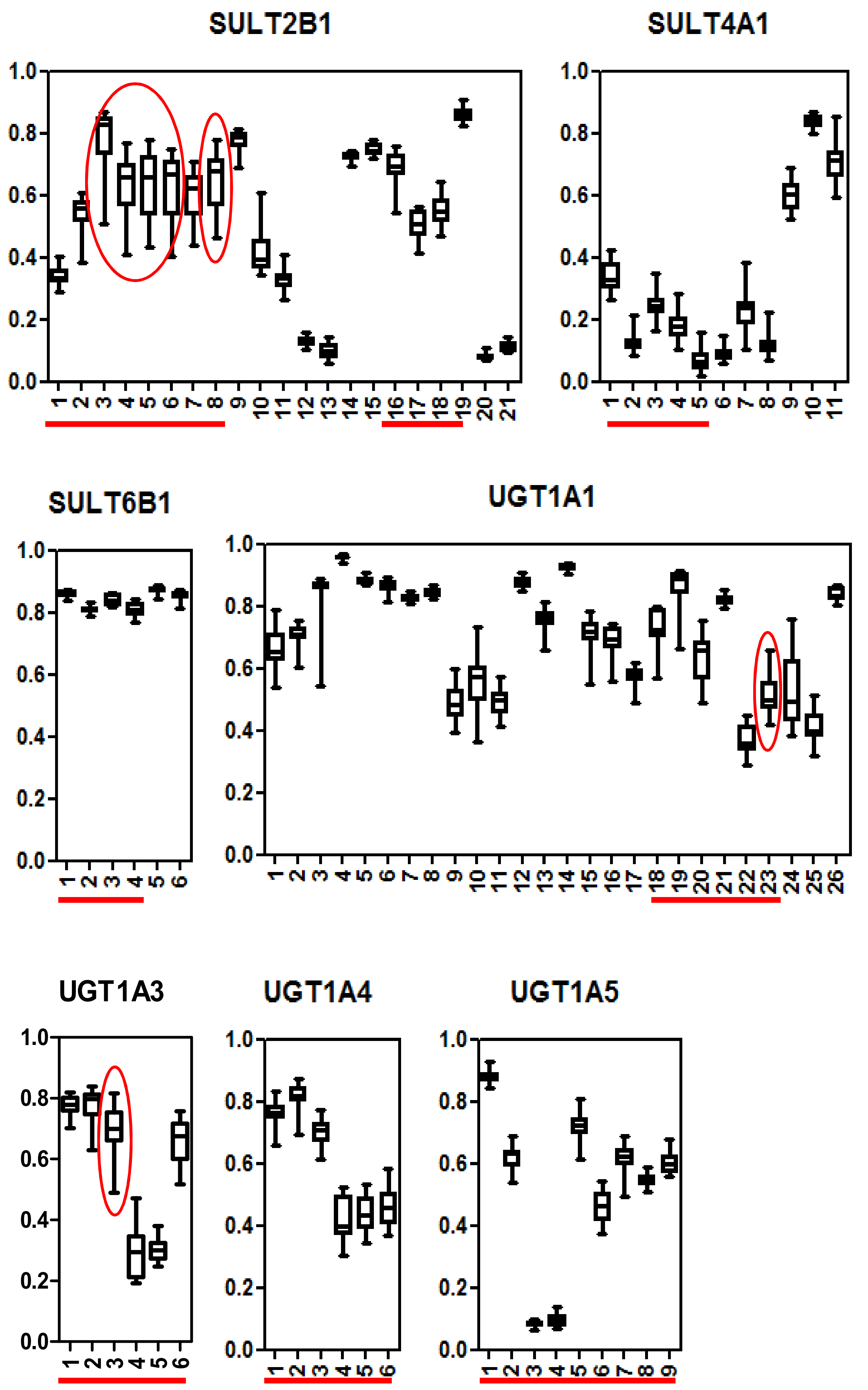

SULT2B1
SULT4A1
SULT6B1
UGT1A1
UGT1A3
UGT1A4
UGT1A5
1.0
0.8
0.6
0.4
0.2
0.0

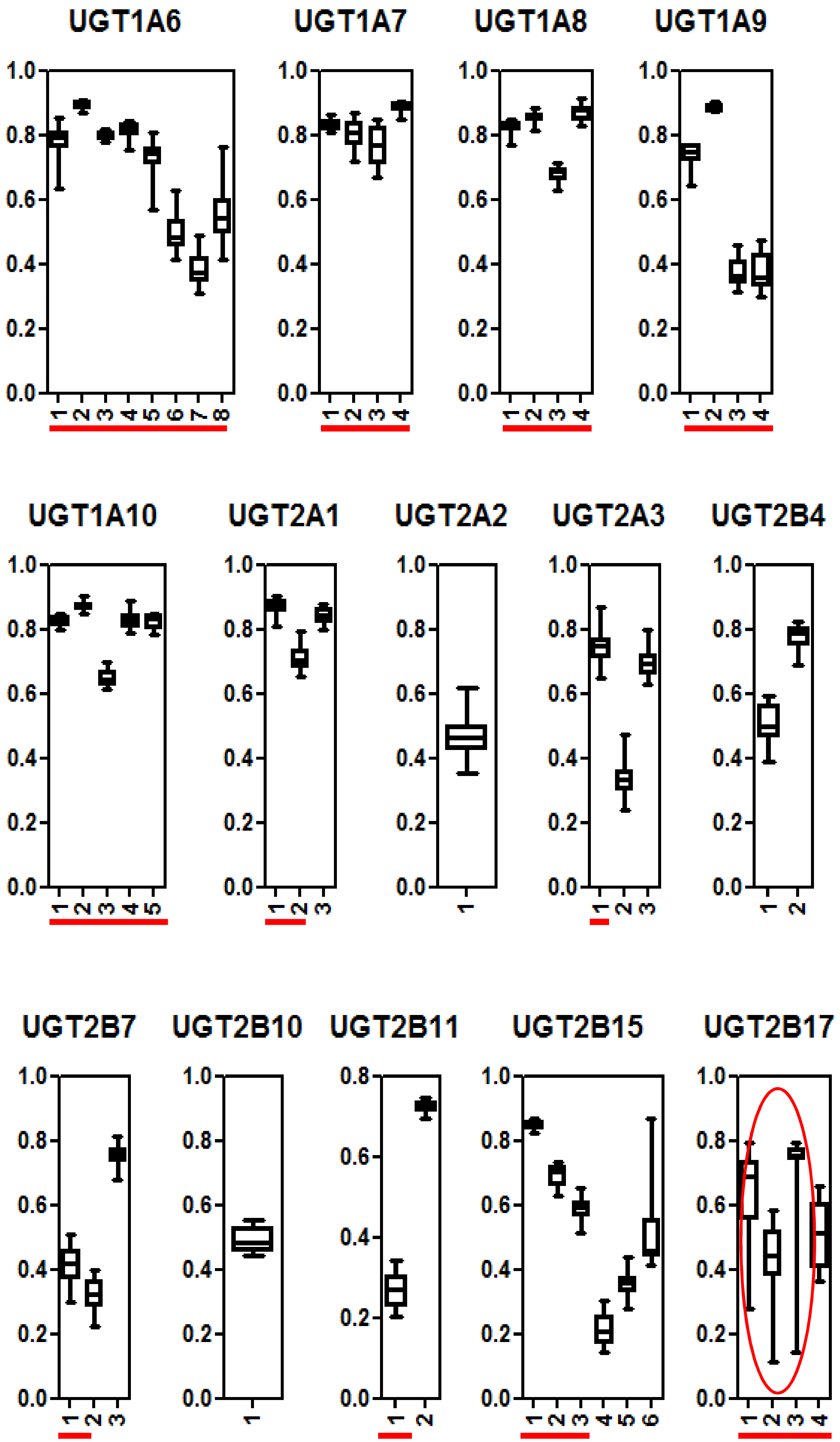

UGT1A6
UGT1A7
UGT1A8
UGT1A9
UGT1A10
UGT2A1
UGT2A2
UGT2A3
UGT2B4
UGT2B7
UGT2B10
UGT2B11
UGT2B15
UGT2B17

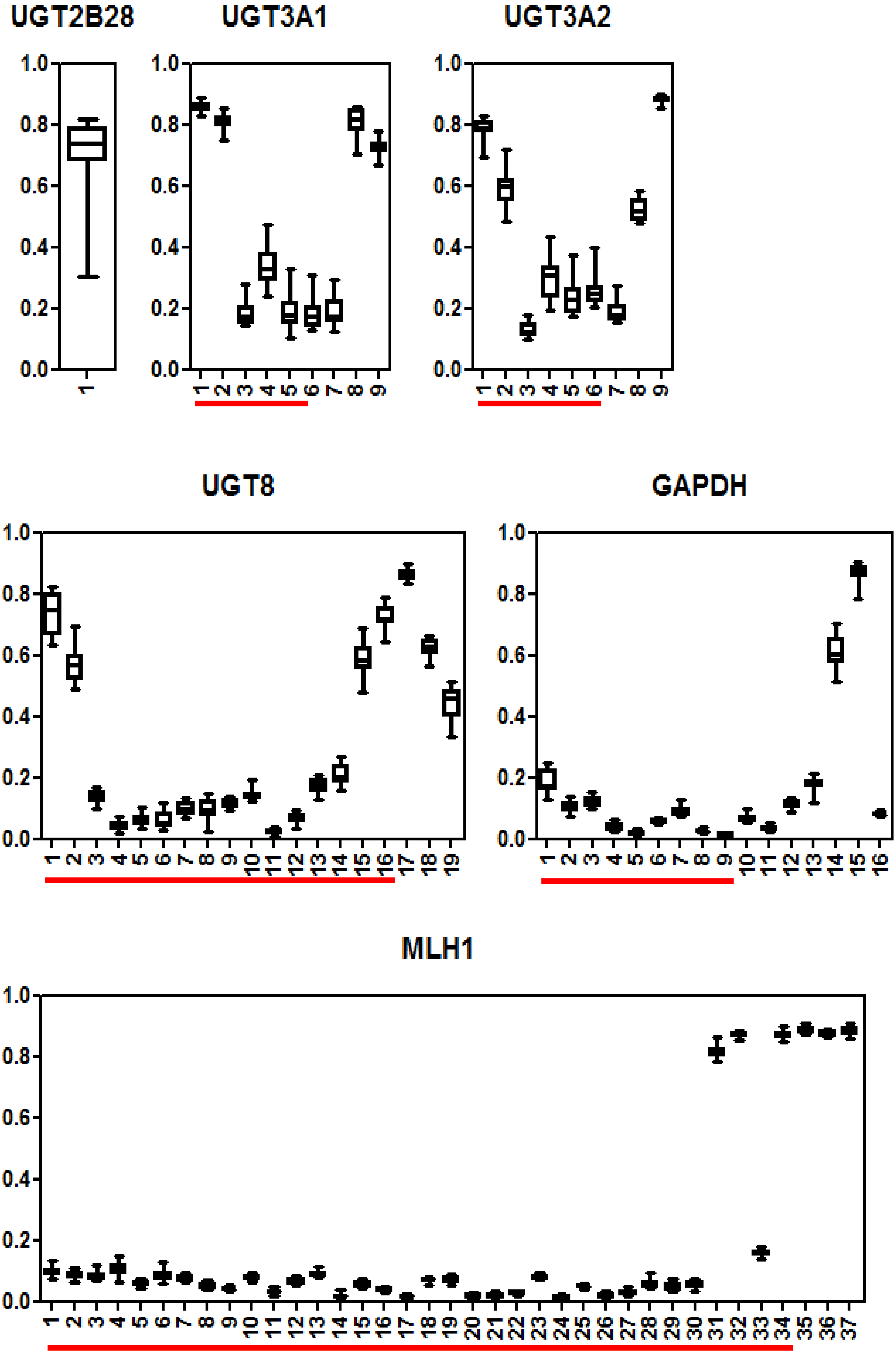
UGT2B28
UGT3A1
UGT3A2
UGT8
GAPDH
MLH1
0.0
0.2
0.4
0.6
0.8
1.0

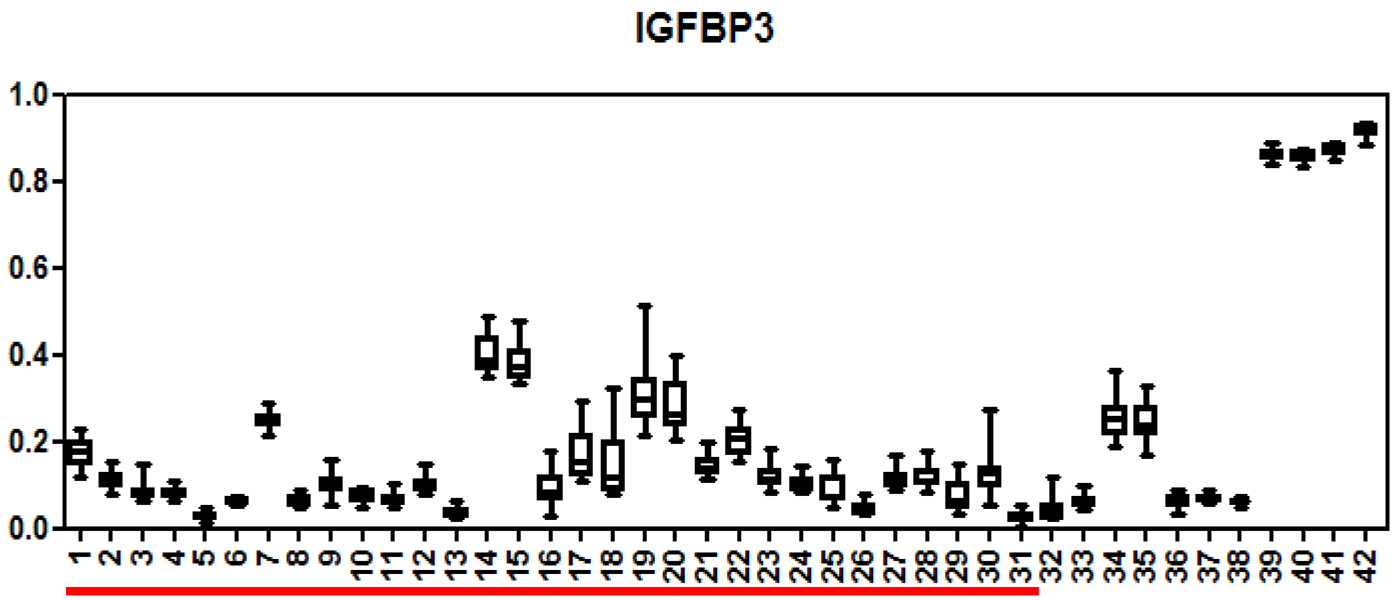

IGFBP3
1.0
0.8
0.6
0.4
0.2
0.0
1 2 3 4 5 6 7 8 9 10 11 12 13 14 15 16 17 18 19 20 21 22 23 24 25 26 27 28 29 30 31 32 33 34 35 36 37 38 39 40 41 42

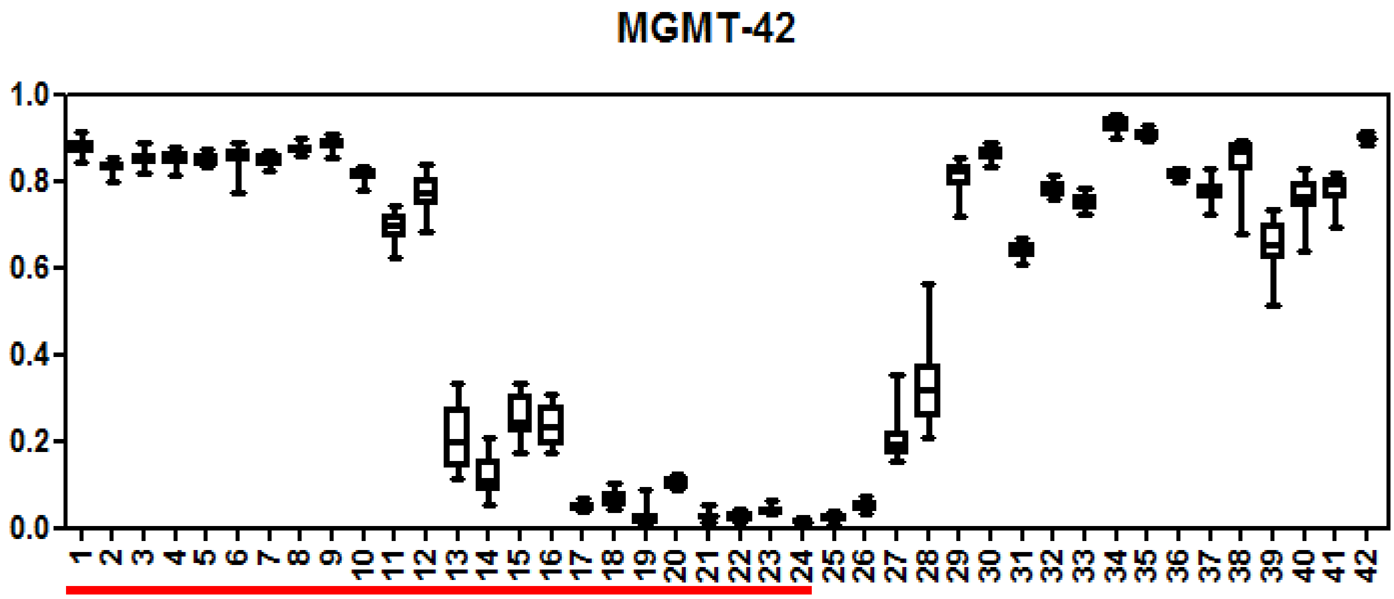

MGMT-42
1.0
0.8
0.6
0.4
0.2
0.0
1 2 3 4 5 6 7 8 9 10 11 12 13 14 15 16 17 18 19 20 21 22 23 24 25 26 27 28 29 30 31 32 33 34 35 36 37 38 39 40 41 42
