# Supplementary figures and images for: Analysis of DNA methylation landscape reveals the roles of DNA methylation in the regulation of drug metabolizing enzymes
Source: Clin Epigenetics. 2015 Sep 28;7:105. doi: 10.1186/s13148-015-0136-7 (PMC4587720; doi:10.1186/s13148-015-0136-7)

A No. of genes

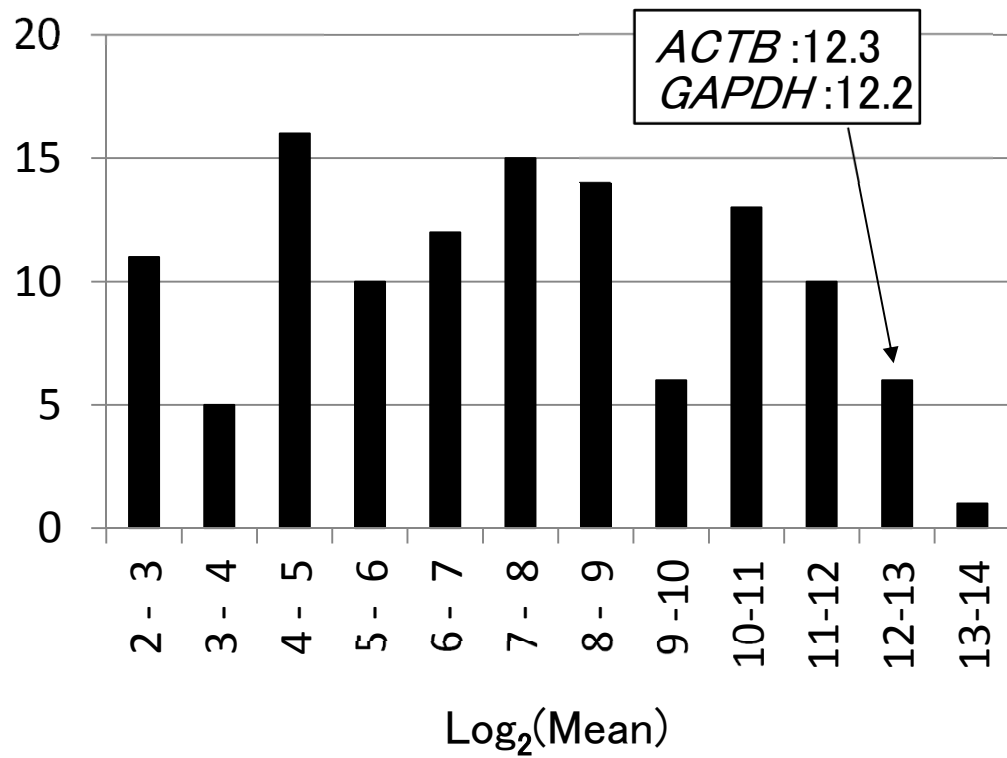

B No. of genes

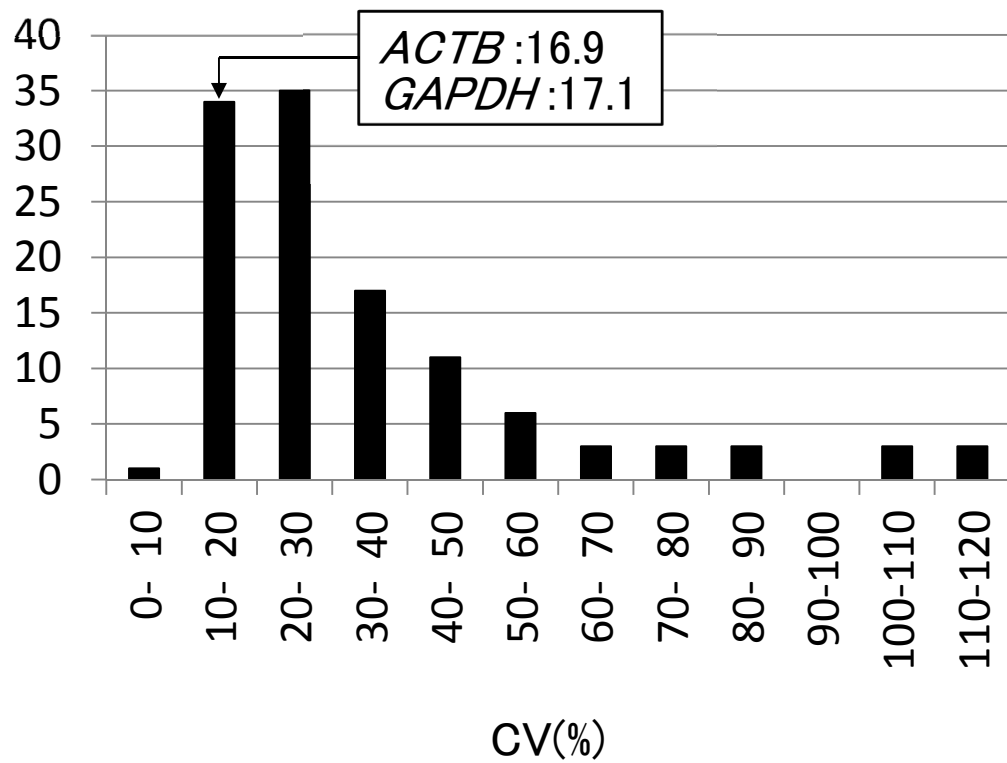

Supplement: Additional file 2: Figure S2. — Distribution of the mRNA expression levels and CV values of DME and control genes. (A) The horizontal axis of the histogram represents the logarithm of the mean value of the transcript (normalized values by the 75th percentile) for 10 liver specimens. (B) The horizontal axis of the histogram represents the CV (%) of the transcript. (PDF 33.6 KB) [file 13148_2015_136_MOESM2_ESM.pdf]

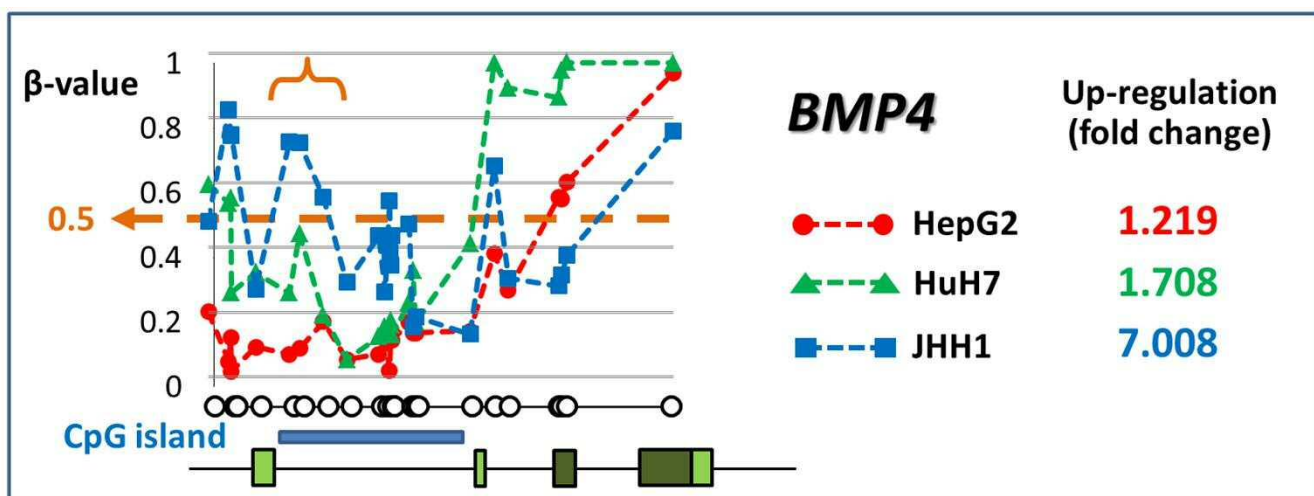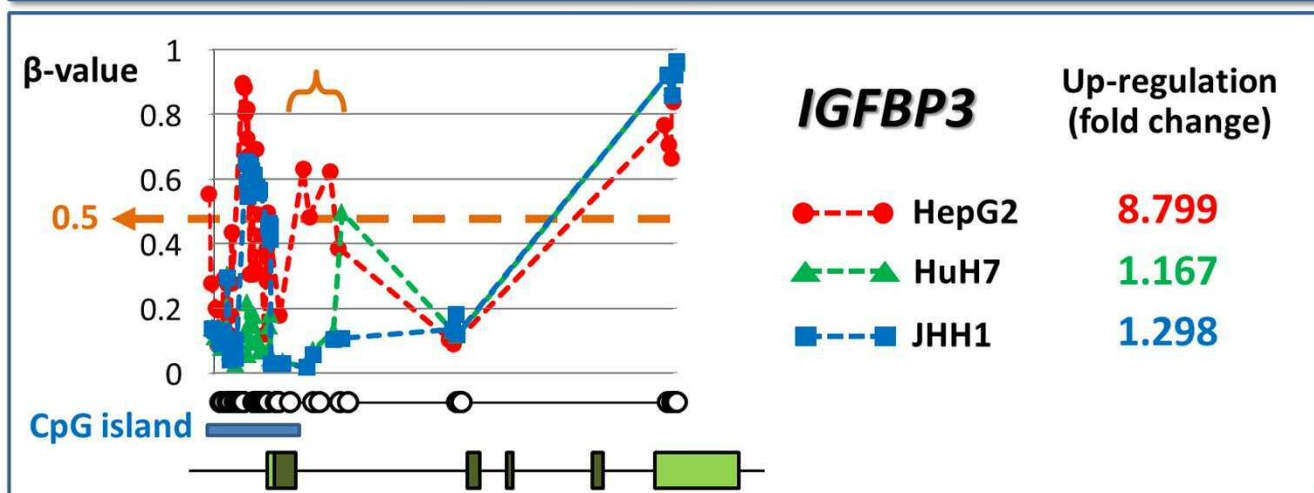

Supplement: Additional file 6: Figure S6. — DNA methylation mapping of BMP4 and IGFBP3 tumor suppressor genes in the three hepatoma cell lines. In each panel, open circles located on the horizontal axis indicate the positions of CpG sites arranged in the 5′ to 3′ direction according to the relative distance of each CpG site. Exons are indicated by green boxes, and translated regions are shown in dark green. The fold change in mRNA expression is shown on the right side of the panel. Following DAC treatment, the BMP4 and IGFBP3 genes were exclusively upregulated in JHH1 (7.0 fold) and HepG2 (8.8 fold) cells, respectively, and hypermethylation was also detected in methylation mapping for these two cell lines, with β values of more than 0.5. (PDF 167 KB) [file 13148_2015_136_MOESM6_ESM.pdf]

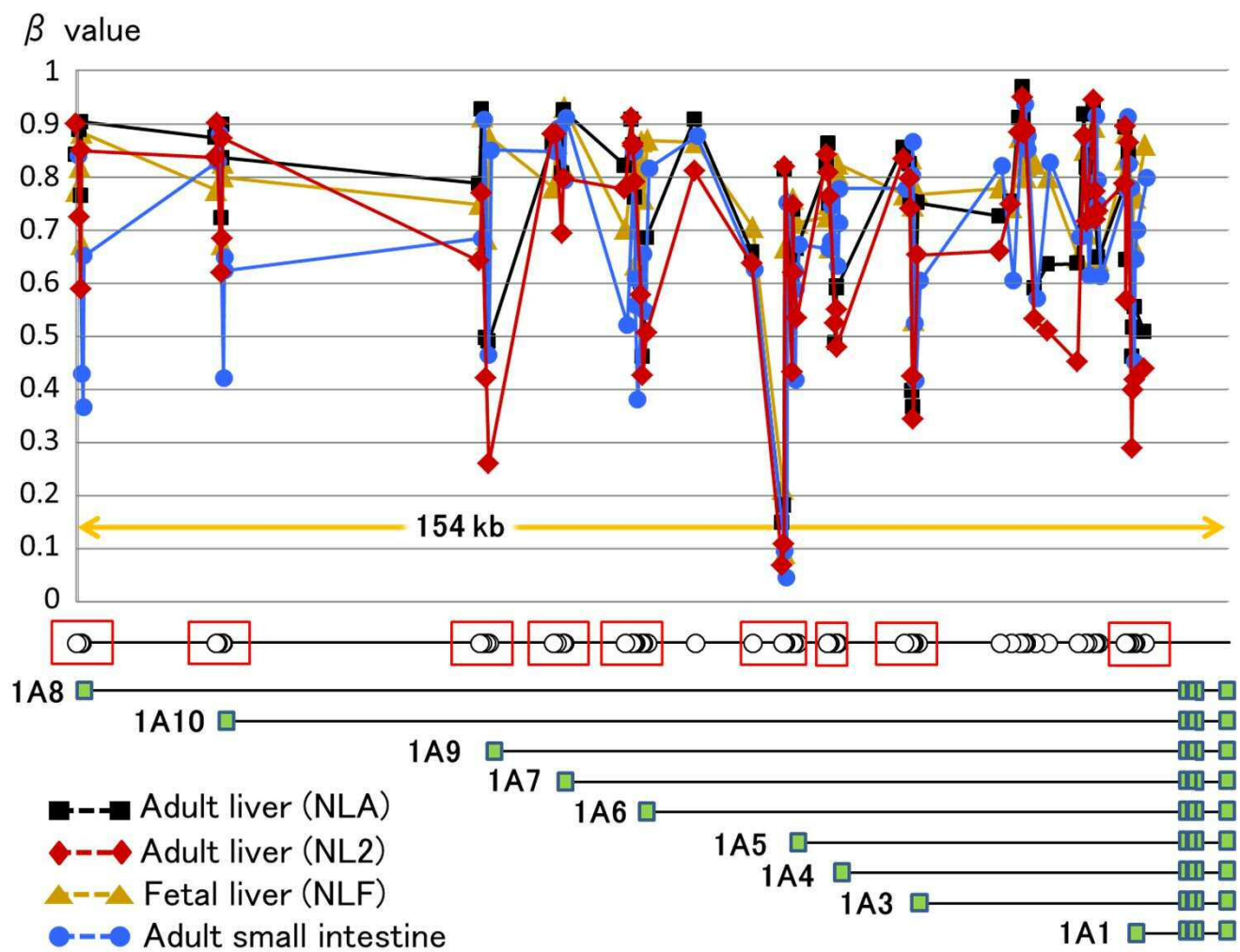

Supplement: Additional file 8: Figure S8. — DNA methylation mapping on the UGT1A locus. The vertical axis of the upper panel shows the level of DNA methylation on the UGT1A locus for different tissues. Open circles on the horizontal axis indicate the positions of CpG sites corresponding to the UGT1A gene structure shown in the lower panel. The UGT1A isoforms are transcribed by an exon sharing mechanism in which the transcripts of the individual first exon cassette are spliced to exons 2–5. The CpG sites boxed in red correspond to the first exon of each isoform. (PDF 179 KB) [file 13148_2015_136_MOESM8_ESM.pdf]
