# Supplementary material for: Analysis of DNA methylation landscape reveals the roles of DNA methylation in the regulation of drug metabolizing enzymes
Source: Clin Epigenetics. 2015 Sep 28;7:105. doi: 10.1186/s13148-015-0136-7 (PMC4587720; doi:10.1186/s13148-015-0136-7)

CYP1A2_#1

Spearman r = −0.6605
P value = 0.0438

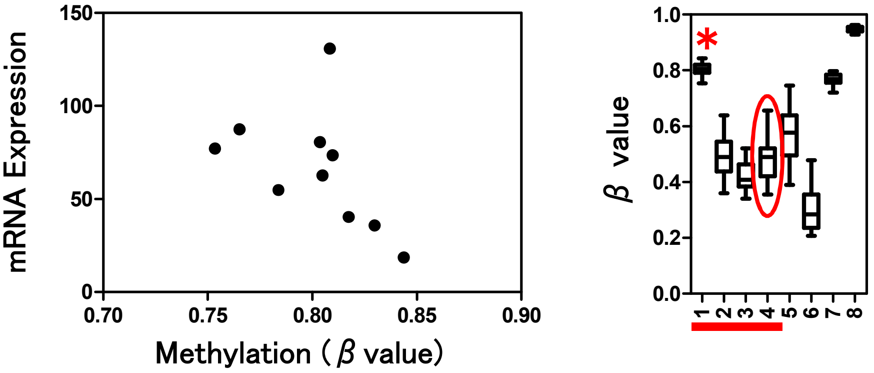

CYP2D6_#2

Spearman r = −0.8303
P value = 0.0047

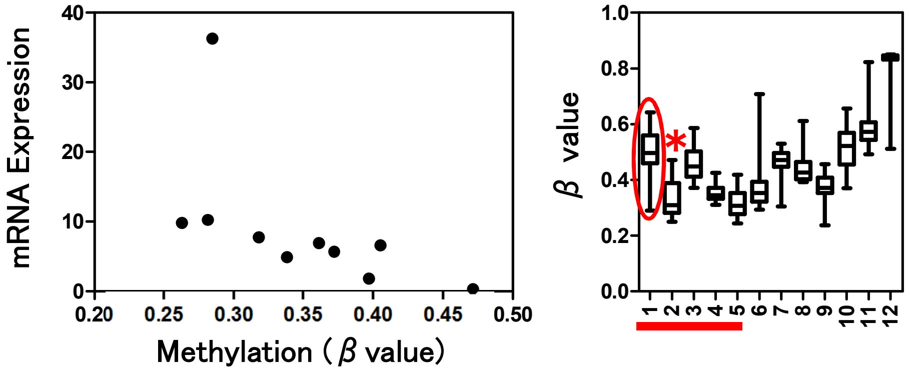

GSTM5_#2

Spearman r = −0.8182
P value = 0.0058

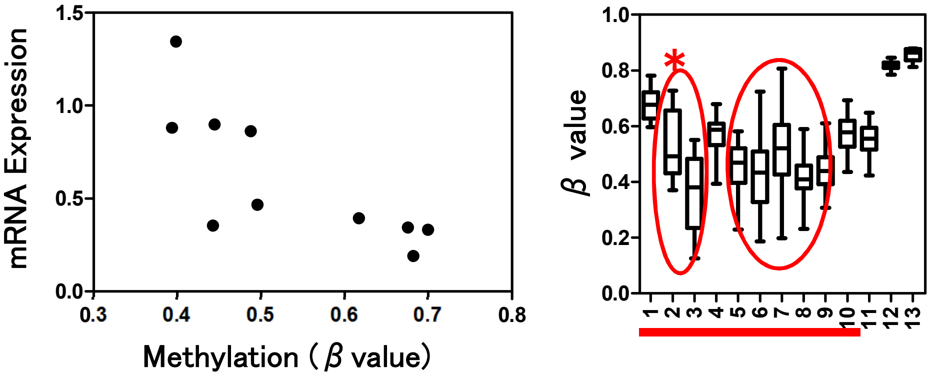

GSTT1_#1

Spearman r = −0.6970
P value = 0.0306

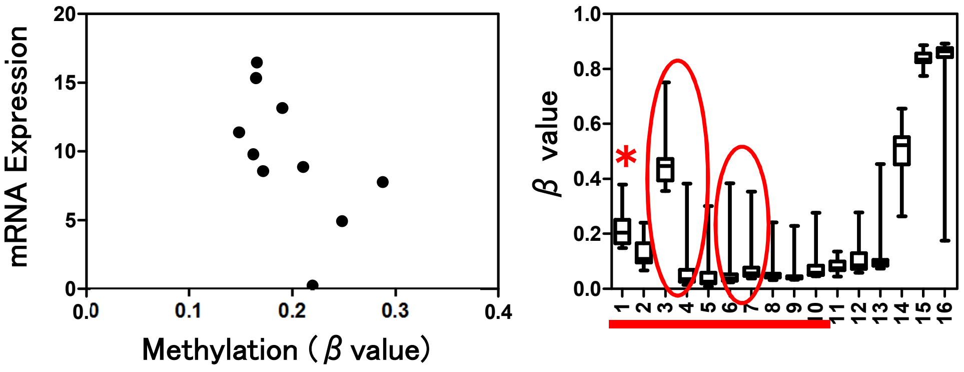

SULT1A1_#11

Spearman r = −0.7697
P value = 0.0126

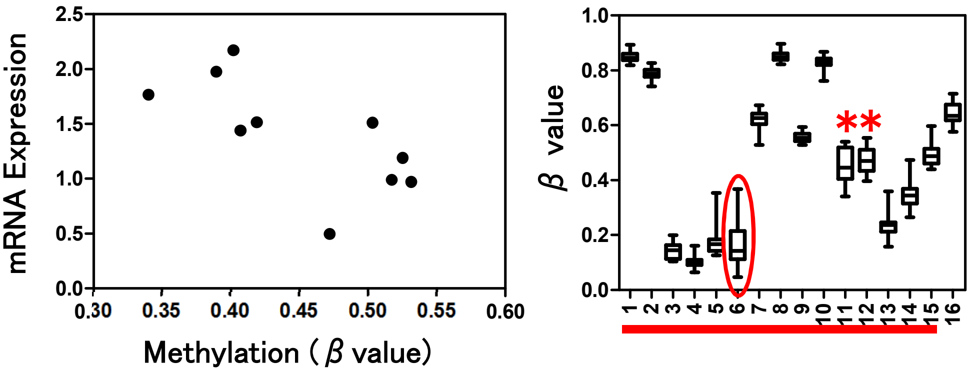

Supplement: Additional file 3: Figure S3. — Representative results of correlation analysis. Significant inverse correlations between DNA methylation levels and mRNA expression levels were detected for the other five DME genes (CYP1A2, CYP2D6, GSTM5, GSTT1, and SULT1A1; p < 0.05, Spearman’s rank correlation test). The mRNA expression level, shown as the vertical axis, was normalized to the level of ACTB mRNA (set as 100 %). CpG sites with significant correlation are indicated by asterisks in the methylation map for each gene. CpG sites showing β R values of more than 0.296 are indicated by circles. (PDF 54.3 KB) [file 13148_2015_136_MOESM3_ESM.pdf]
