# Supplementary material for: Analysis of DNA methylation landscape reveals the roles of DNA methylation in the regulation of drug metabolizing enzymes
Source: Clin Epigenetics. 2015 Sep 28;7:105. doi: 10.1186/s13148-015-0136-7 (PMC4587720; doi:10.1186/s13148-015-0136-7)
Supplement: Additional file 4: Figure S4. — DNA methylation profiles of DME genes in three hepatoma cell lines. DME genes with hypermethylated or hypomethylated CpG sites are listed and shown by closed and shaded boxes, respectively. The number of CpG sites showing hypermethylation or hypomethylation is also indicated in each box. (PDF 31.9 KB) [file 13148_2015_136_MOESM4_ESM.pdf]

Gene	HepG2	HuH7	JHH1
CYP1A1	6	9	11
CYP1A2			1
CYP1B1	14	1	28
CYP2A7	1		
CYP2A13	3	3	
CYP2C8	1	2	2
CYP2C18	1	1	
CYP2D6			1
CYP2F1	5	3	
CYP2W1	6		
CYP3A43	1	1	
CYP4F2	1		
CYP4F3			1
CYP4F8	1		1
CYP4F11		1	
CYP4X1	1	2	
CYP7B1	3		2*
CYP8B1		1	
CYP11A1			4
CYP11B2	1	1	1
CYP19A1	4*	4	
CYP20A1			1
CYP21A2		1	
CYP24A1	6	2	
CYP26C1	2	4	3
CYP27B1	2		

Gene	HepG2	HuH7	JHH1
GSTA2		1	
GSTA4	1		
GSTM1	1		2
GSTM2			1
GSTM3	2		
GSTM4	1		
GSTM5			3
GSTO2	11	13	
GSTP1	4		
NAT1	1	1	1
NAT8L			1
NAT15		1	
SULT1A1	2	1	
SULT1B1	1	1	1
SULT1C2			1
SULT1C4			1
SULT2B1	4		
UGT1A1			1
UGT1A6			3
UGT2A1	1	2	2
UGT2A3	1	1	1
UGT2B7	1	1	1
UGT2B15	4	3	1
UGT3A1		1	2
UGT3A2		4	
UGT8	9		
