# Supplementary material for: Analysis of DNA methylation landscape reveals the roles of DNA methylation in the regulation of drug metabolizing enzymes
Source: Clin Epigenetics. 2015 Sep 28;7:105. doi: 10.1186/s13148-015-0136-7 (PMC4587720; doi:10.1186/s13148-015-0136-7)
Supplement: Additional file 5: Figure S5. — Changes in the mRNA expression levels of DME genes in three hepatoma cell lines after DAC treatment. DME genes showing upregulation in at least one hepatoma cell line (greater that twofold change) following DAC treatment are listed. The fold change is indicated in each box, and genes with fold changes of more than two are indicated by closed boxes. The six DME genes exhibiting correlations between mRNA expression and DNA methylation status are shown in highlighted boxes. (PDF 37.9 KB) [file 13148_2015_136_MOESM5_ESM.pdf]

Gene	HepG2	HuH7	JHH1
CYP1A2	2.5	2.6	3.2
CYP1B1	1.3	4.2	110.5
CYP2B6	2.8	0.7	1.2
CYP2C9	0.8	1.3	2.1
CYP2C18	1.3	3.8	1.6
CYP2C19	0.8	0.7	3.5
CYP2F1	25.1	1.5	3.6
CYP2J2	1.8	0.9	2.1
CYP2R1	15.3	1.2	1.0
CYP2S1	2.3	2.0	2.8
CYP3A5	5.8	0.7	0.8
CYP3A7	6.2	0.5	0.6
CYP3A43	2.0	1.0	0.6
CYP4F3	1.6	0.5	2.2
CYP4F22	0.8	1.0	5.0
CYP4V2	2.7	1.1	0.5
CYP7B1	1.0	4.6	0.8
CYP8B1	1.4	2.6	0.9
CYP11A1	2.2	2.1	1.4
CYP19A1	2.0	0.5	0.8
CYP24A1	4.0	0.8	10.0
CYP26A1	2.4	10.7	3.1
CYP26B1	2.3	1.5	1.4
CYP26C1	3.4	1.4	0.9
CYP27B1	2.5	3.4	4.2
CYP27C1	0.8	4.9	0.8
CYP46A1	0.8	3.2	0.8

Gene	HepG2	HuH7	JHH1
GSTA2	0.4	3.8	2.1
GSTA5	0.5	4.4	1.8
GSTM2	0.6	1.3	2.1
GSTO2	4.4	1.1	0.6
GSTP1	624.8	0.8	1.0
GSTT2	2.1	1.0	2.1
GSTT2B	2.4	1.1	1.0
NAT8	2.1	0.4	0.8
NAT8B	2.0	0.5	1.0
SULT1C4	4.5	1.2	0.8
SULT2B1	18.0	4.7	3.7
SULT4A1	4.9	0.4	1.0
UGT1A6	5.7	2.2	0.9
UGT1A8	3.4	2.3	1.0
UGT2B15	7.1	2.0	0.8
UGT3A2	1.4	3.2	1.3
UGT8	2.1	3.7	0.5
