# Supplementary material for: Analysis of DNA methylation landscape reveals the roles of DNA methylation in the regulation of drug metabolizing enzymes
Source: Clin Epigenetics. 2015 Sep 28;7:105. doi: 10.1186/s13148-015-0136-7 (PMC4587720; doi:10.1186/s13148-015-0136-7)
Supplement: Additional file 7: Figure S7. — Expression levels of UGT1A transcripts in different tissues. The vertical axis indicates the mRNA expression levels of UGT1A isoforms normalized to the level of UGT1A1 expression in adult liver (NL2; set as 100 %). Each column represents the mean ± SD (n = 3). (PDF 35.5 KB) [file 13148_2015_136_MOESM7_ESM.pdf]

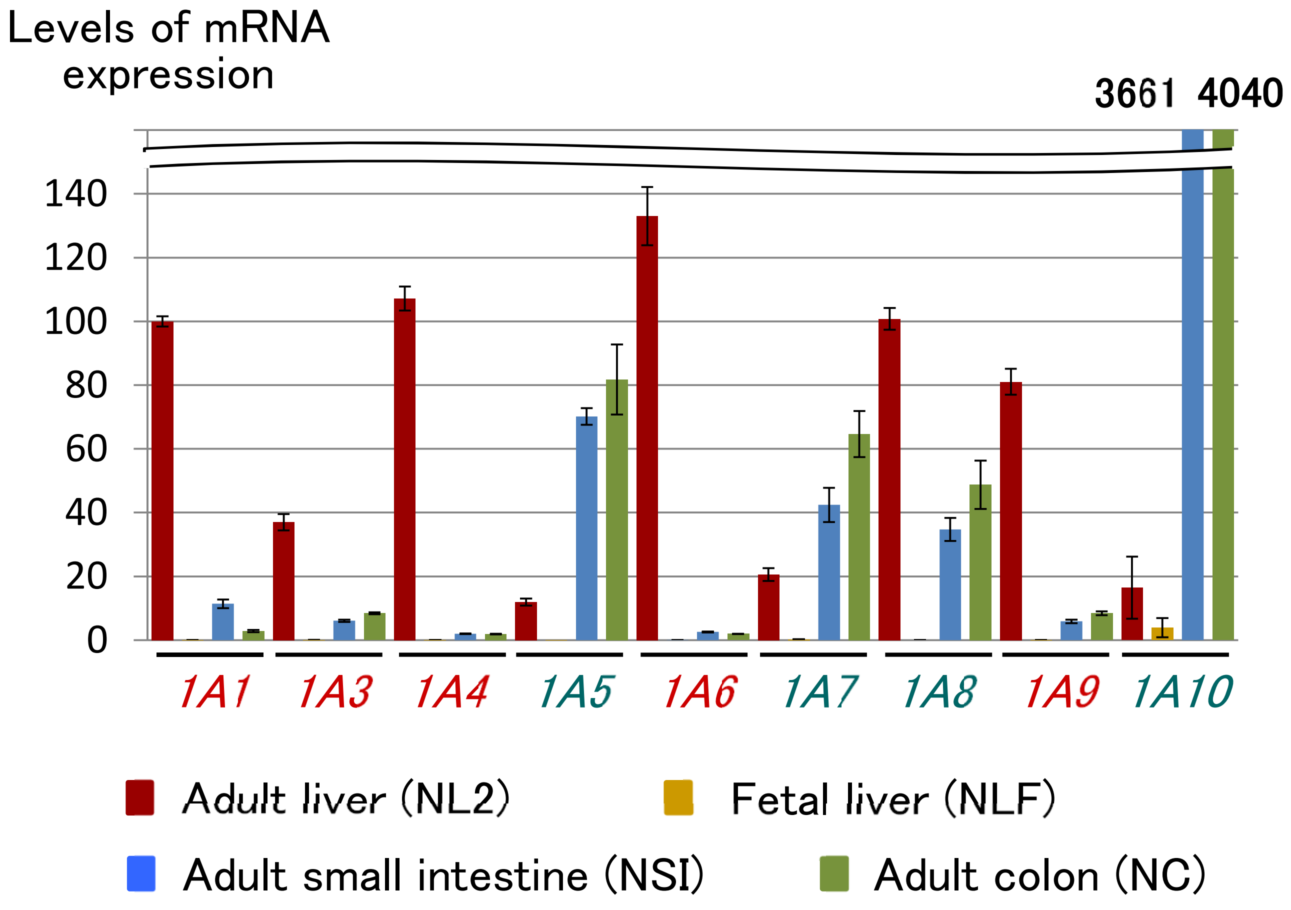

Hepatic type; *UGT1A1*, *UGT1A3*, *UGT1A4*, *UGT1A6*, *UGT1A9*

Intestinal type; *UGT1A5*, *UGT1A7*, *UGT1A8*, *UGT1A10*
