# Supplementary material for: Analysis of DNA methylation landscape reveals the roles of DNA methylation in the regulation of drug metabolizing enzymes
Source: Clin Epigenetics. 2015 Sep 28;7:105. doi: 10.1186/s13148-015-0136-7 (PMC4587720; doi:10.1186/s13148-015-0136-7)
Supplement: Additional file 10: Table S2. — Datasets deposited in the GEO repository. (DOC 46.5 KB) [file 13148_2015_136_MOESM10_ESM.doc]

Table S1. Datasets deposited in the GEO repository.

| Series  /Platforms | Sample name | Title | Source name | Samples |
| --- | --- | --- | --- | --- |
| GSE67477  /[GPL13534](http://www.ncbi.nlm.nih.gov/geo/query/acc.cgi?acc=GPL13534) | BA0002_01_a | HepG2_C | HepG2 | GSM[1647842](http://www.ncbi.nlm.nih.gov/geo/query/acc.cgi?acc=GSM1647842)^a^ |
|  | BA0002_02_a | HepG2_T | HepG2, TSA treated | * |
|  | BA0002_03_a | HuH7_C | HuH7 | GSM[1647844](http://www.ncbi.nlm.nih.gov/geo/query/acc.cgi?acc=GSM1647844)^b^ |
|  | BA0002_04_a | JHH1_C | JHH1 | GSM[1647845](http://www.ncbi.nlm.nih.gov/geo/query/acc.cgi?acc=GSM1647845)^c^ |
|  | BA0002_05_a | JHH1_T | JHH1, TSA treated | * |
|  | BA0002_06_a | NLA | liver tissue, adult | GSM[1647847](http://www.ncbi.nlm.nih.gov/geo/query/acc.cgi?acc=GSM1647847) |
|  | BA0002_07_a | NLF | liver tissue, fetal | GSM[1647848](http://www.ncbi.nlm.nih.gov/geo/query/acc.cgi?acc=GSM1647848) |
| GSE67484  /[GPL13534](http://www.ncbi.nlm.nih.gov/geo/query/acc.cgi?acc=GPL13534) | L_1 | L_1 | liver tissue, adult | * |
|  | SI_1 | SI_1 | small intestine tissue, adult | * |
|  | L_2 | L_2 | liver tissue, adult | GSM[1647887](http://www.ncbi.nlm.nih.gov/geo/query/acc.cgi?acc=GSM1647887)^d^ |
|  | SI_2 | SI_2 | small intestine tissue, adult | GSM[1647888](http://www.ncbi.nlm.nih.gov/geo/query/acc.cgi?acc=GSM1647888)^e^ |
| GSE67318  /[GPL17077](http://www.ncbi.nlm.nih.gov/geo/query/acc.cgi?acc=GPL17077) | 1C | no1 1C | HepG2 | GSM[1644679](http://www.ncbi.nlm.nih.gov/geo/query/acc.cgi?acc=GSM1644679)^a^ |
|  | 1D | no2 1D | Hepg2, treated with DAC | GSM[1644680](http://www.ncbi.nlm.nih.gov/geo/query/acc.cgi?acc=GSM1644680) |
|  | 2C | no3 2C | HuH7 | GSM[1644681](http://www.ncbi.nlm.nih.gov/geo/query/acc.cgi?acc=GSM1644681)^b^ |
|  | 2D | no4 2D | HuH7, treated with DAC | GSM[1644682](http://www.ncbi.nlm.nih.gov/geo/query/acc.cgi?acc=GSM1644682) |
|  | 3C | no5 3C | JHH1 | GSM[1644683](http://www.ncbi.nlm.nih.gov/geo/query/acc.cgi?acc=GSM1644683)^c^ |
|  | 3D | no6 3D | JHH1, treated with DAC | GSM[1644684](http://www.ncbi.nlm.nih.gov/geo/query/acc.cgi?acc=GSM1644684) |
|  | L | no7 L | liver adult | GSM[1644685](http://www.ncbi.nlm.nih.gov/geo/query/acc.cgi?acc=GSM1644685)^d^ |
|  | SI | no8 SI | small intestine, adult | GSM[1644686](http://www.ncbi.nlm.nih.gov/geo/query/acc.cgi?acc=GSM1644686)^e^ |

*Data samples not used for the present study.

^a, b, c, d^ or ^e^; Paired DNA methylation and mRNA expression data samples derived from the same specimens.
