# Supplementary material for: Analysis of DNA methylation landscape reveals the roles of DNA methylation in the regulation of drug metabolizing enzymes
Source: Clin Epigenetics. 2015 Sep 28;7:105. doi: 10.1186/s13148-015-0136-7 (PMC4587720; doi:10.1186/s13148-015-0136-7)
Supplement: Additional file 11: Table S3. — Primers used for mRNA expression and DNA methylation analyses. (DOC 44.5 KB) [file 13148_2015_136_MOESM11_ESM.doc]

Table S3. Primers used for mRNA expression and DNA methylation analyses.

| Gene | | Primer sequence (5’-3’) | Product  size (bp) |
| --- | --- | --- | --- |
| *CYP1B1* | E_Forward  E_Reverse | CGGCTGGATTTGGAGAACGTA  TGATCCAATTCTGCCTGCACT | 146 |
|  | M_Forward  M_Reverse | GGTTTTTTTATAAAGGGAGGGTTT  ACTAAAAAAACCTAAAAAAACTAAC | 480 |
| *CYP8B1* | E_Forward  E_Reverse | TCATTGCTGGATACCTGTGC  GGTCCATGACGAAGGTGAAG | 207 |
|  | M_Forward  M_Reverse | GAGTAGTTGTGATTATAGTTGGAA  CCAAAAAACCATAACTATACTCCT | 288 |
| *GSTM2* | E_Forward  E_Reverse | CCAGAGCAACGCCATCCT  GATTCCCCGCACAGGTTGT | 57 |
|  | M_Forward  M_Reverse | ggaggtgggaggagattttat  tcctacaactactccacacttcc | 298 |
| *GSTP1* | E_Forward  E_Reverse | ACCTCCGCTGCAAATACATC  GACAGCAGGGTCTCAAAAGG | 109 |
|  | M_Forward  M_Reverse | ttggggatttgggaaagagggaa  ttaaccccatactaaaaactcta | 318 |
| *UGT3A2* | E_Forward  E_Reverse | CATATCAAGTTATCAGTTGGCTTG  ACTGCACTGCAACGCCAAGTA | 149 |
|  | M_Forward  M_Reverse | taggtaggagggttgtgattata  tacccaaccatactcacttcta | 301 |
| *ACTB* | E_Forward  E_Reverse | TCATGAAGTGTGACGTGGACATC  CAGGAGGAGCAATGATCTTGATCT | 156 |
| *UGT1A1 ** | | Hs02511055_s1 |  |
| *UGT1A3* * | | Hs04194492_g1 |  |
| *UGT1A4 ** | | Hs01655285_s1 |  |
| *UGT1A5 ** | | Hs01374521_s1 |  |
| *UGT1A6 ** | | Hs01592477_m1 |  |
| *UGT1A7 ** | | Hs02517015_s1 |  |
| *UGT1A8 ** | | Hs01592482_m1 |  |
| *UGT1A9 ** | | Hs02516855_sH |  |
| *UGT1A10 ** | | Hs02516990_s1 |  |
| *ACTB ** | | Hs99999903_m1 |  |

E, primer used for mRNA expression analysis (quantitative real-time PCR)

M, primer used for DNA methylation analysis (COBRA)

*TaqMan probe sets
